# Application and Single‐Cell Regulation Mechanism of Engineered EVs Combined With 4D‐Printed Hydrogel for Infected Burn Repair

**DOI:** 10.1002/advs.77141

**Published:** 2026-08-21

**Authors:** Xiaomin Wang, Xiaoyan Li, Xiao Xu, Kelun Zhang, Fengjiao Yu, Zhen Shang, Nailong Pan, Junlin Lv, Yiwei Xu, Yan Tang, Liang Zhang, Xiaotong Li, Taoyi Wang, Jing Tao, Jiaxu Song, Mengyu Zhang, Xin Ma, Dan Han, Xiaoying Kong, Wenhua Xu

**Affiliations:** ^1^ Institute of Regenerative Medicine and Laboratory Technology Innovation Qingdao University Qingdao Shandong P. R. China; ^2^ School of Basic Medicine Qingdao University Qingdao P. R. China; ^3^ Department of Dermatology and Cutaneous Biology Research Institute Severance Hospital Yonsei University College of Medicine Seoul South Korea

**Keywords:** 4D biopriting, burns, cells crosstalk, engineered EVs, FGF2

## Abstract

Burns represent a complex and disabling global public health problem, presenting substantial clinical challenges attributed to disrupted tissue microenvironment and persistent wound infections, which impair the synergistic interaction between fibroblasts (Fb) and keratinocytes (KC). FGF2 and its homologues widely used for burns show weak wound‐repair efficacy, owing to poor targeting and degradation by bacteria and proteases. In this study, FGF2‐modified extracellular vesicles (F‐EVs) were engineered from human adipose mesenchymal stem cells (ADMSCs) via electrostatic adsorption, and further combined with 4D‐printed hydrogels to construct a 4D@F‐EVs system. This system was designed to reconstruct the burn tissue microenvironment, exert antibacterial effects, and restore the crosstalk between Fb and KC. In vitro and in vivo analysis confirmed that the 4D@F‐EVs were precisely delivered to Fb, promoting cell proliferation and facilitating the reconstruction of the burn tissue microenvironment. Single‐cell RNA sequencing (scRNA‐seq) analysis revealed that the regulatory effect of 4D@F‐EVs on skin regeneration involves the activation of the PI3K‐Akt signaling pathway and the promotion of crosstalk between Keratinocyte Growth Factor‐positive (KGF^+^) Fb and Keratinocyte Growth Factor Receptor‐positive (KGFR^+)^ KC. Collectively, this work offers an original clinical approach for infected burn wounds by reconstructing the impaired microenvironment and restoring intercellular crosstalk.

## Graphical abstract

4D bioprinted hydrogel loaded with FGF2‐modified extracellular vesicles (4D@F‐EVs) targets dermal fibroblasts and elevates local wound FGF2 levels via sustained release of F‐EVs. It activates PI3K‐Akt signaling, restores critical KGF^+^ fibroblast–KGFR^+^ keratinocyte crosstalk, enhances robust angiogenesis and collagen deposition to efficiently repair damaged infected burn wounds.

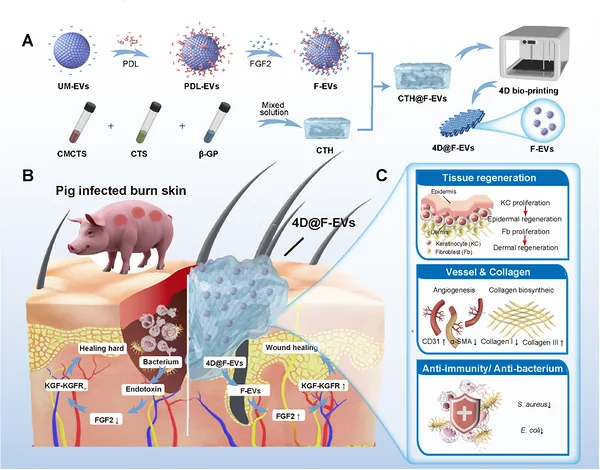

**Abbreviations**
4D‐bioprinted thermo‐responsive chitosan‐based hydrogel: 4D‐CTH
4D‐printed hydrogel loaded With F‐EVs: 4D@F‐EVs
Adipose‐derived mesenchymal stem cells: ADMSCs
Alpha‐smooth muscle actin: α‐SMA
Chitosan/carboxymethyl chitosan/β‐glycerophosphate thermosensitive hydrogel: CTH
Cluster of differentiation 31: CD31
Carboxymethyl chitosan: CMCTS
Chitosan: CTS
Dynamic light scattering: DLS
Extracellular vesicles: EVs
FGF2‐modified extracellular vesicles: F‐EVs
Fibroblast growth factor 2: FGF2
Fibroblasts: Fb
Fourier‐transform infrared spectroscopy: FTIR
Hematoxylin and eosin: H&E
Keratinocyte Growth Factor positive: KGF^+^
Keratinocyte Growth Factor Receptor positive: KGFR^+^
Keratinocyte growth factor receptor: KGFR
Keratinocyte growth factor: KGF
Keratinocytes: KC
Poly‐D‐lysine: PDL
Phosphoinositide 3‐kinase: PI3K
Poly‐D‐lysine‐modified extracellular vesicles: PDL‐EVs
Recombinant human basic fibroblast growth factor: rhFGF2
Scanning electron microscopy: SEM
Single‐cell RNA sequencing: scRNA‐seq
Transmission electron microscopy: TEM
Unmodified extracellular vesicles: UM‐EVs
β‐Glycerophosphate disodium salt: β‐GP

## 1 Introduction

Burns are sudden, complex and highly disabling global public health issues [1], primarily characterized by severe damage to fibroblasts (Fb) and keratinocytes (KC) as well as impaired crosstalk between these cells [2]. This leads to structural and functional damage in wounds tissue, increased infection risk and scar formation, causing delayed healing and significant clinical challenges [3, 4, 5, 6]. Developing effective new strategies for burn treatment that focus on structural reconstruction and functional repair remains a challenging yet strategically important topic.

Currently, clinical treatments for burns mainly involve early surgical debridement of necrotic tissue, skin grafting and local application of antimicrobial agents. However, these approaches often fall short due to their inability to induce a dynamic, coordinated multicellular process [7]. Studies have shown that basic fibroblast growth factor (bFGF), also referred to as fibroblast growth factor 2 (FGF2), is utilized in the burn wound repair owning to its significant ability to promote the proliferation of various cells (e.g., Fb and KC) and enhance the intercellular crosstalk [8, 9, 10, 11, 12]. Building on the proven efficacy of FGF2, a series of related therapeutics—most notably the recombinant human FGF2 (rhFGF2) hydrogel—have been developed [13, 14]. However, their non‐targeted design and susceptibility to degradation limit their therapeutic efficacy [15, 16].

Nanocarrier‐based strategies hold great promise in addressing the two key limitations of FGF2 in burn repair, namely insufficient targeting and poor stability, via precise delivery and microenvironment‐protective effects [17, 18]. Extracellular vesicles (EVs) serve as natural carriers capable of efficiently transporting bioactive molecules to target cells, thereby playing a critical role in tissue remodeling and intercellular crosstalk following injury [19, 20]. Nevertheless, biosafety concerns associated with engineered EVs have restricted their further clinical translation [21]. In addition, conventional hydrogel scaffolds typically exhibit irregular pore structures, which not only fail to provide a stable and favorable niche for EV retention but also induce local EV aggregation and accelerated inactivation [22, 23]. Therefore, the rational design of safe and high‐performance engineered EV–integrated scaffolds for the effective delivery of FGF2 is of great significance to facilitate burn wound tissue repair.

**FIGURE 1:**
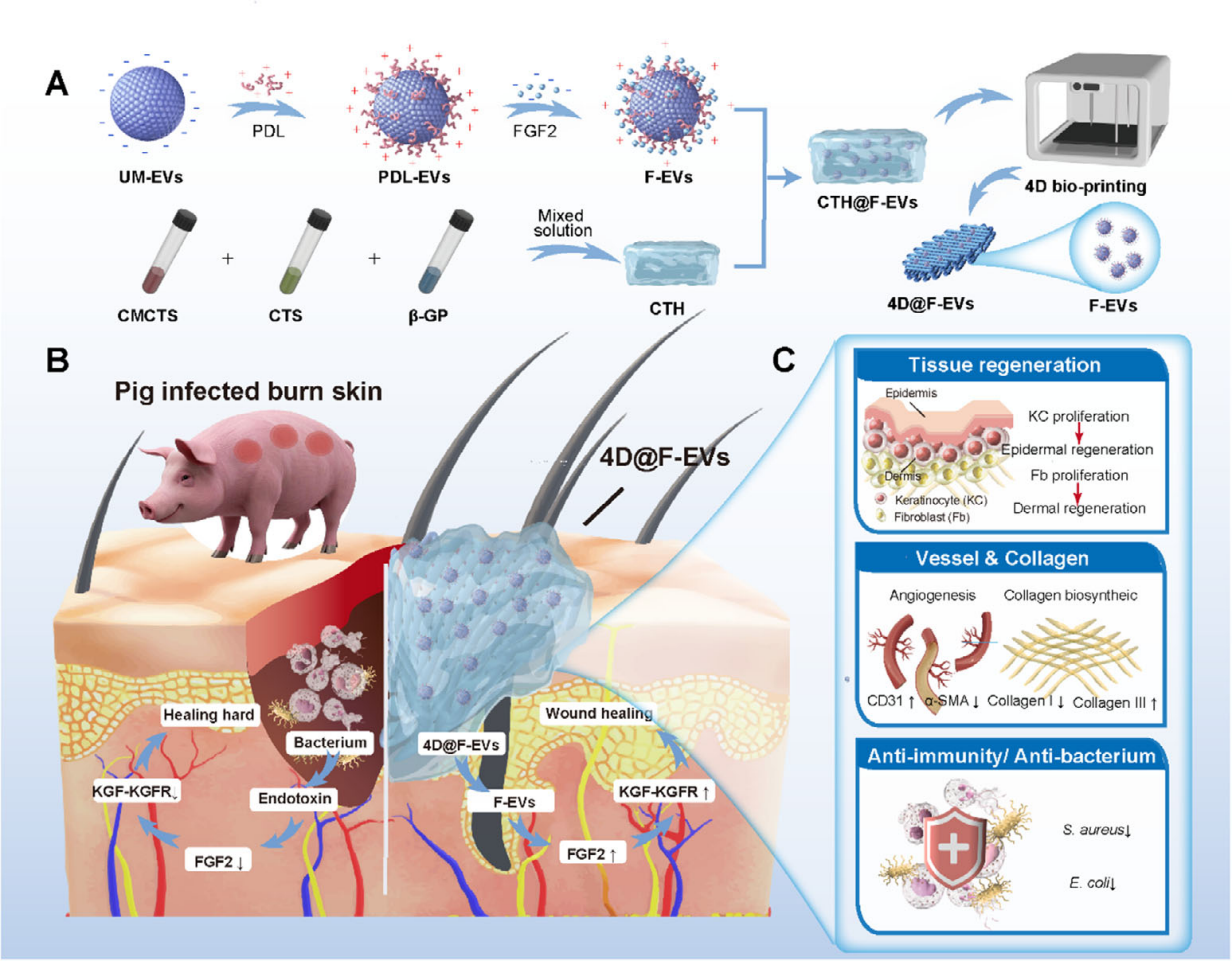
4D@F‐EVs promotes burn wound healing by regulating KGF^+^ Fb and KGFR^+^ KC. (A) Preparation of engineered EVs and 4D printed scaffold. (B) 4D@F‐EVs up‐regulate FGF2 expression in Fb by releasing F‐EVs, modulate KGF‐KGFR interactions, promote KC proliferation, and enhance Fb‐KC crosstalk to accelerate repair. (C) 4D@F‐EVs drive tissue regeneration, enhance collagen maturation and angiogenesis, and exert antimicrobial effects, collectively promoting wound healing. CTS: Chitosan; CMCTS: Carboxymethyl chitosan; β‐GP: β‐Glycerophosphate disodium salt; PDL: Poly‐D‐lysine; Unmodified EVs: UM‐EVs; PDL‐modified EVs: PDL‐EVs; FGF2‐modified EVs: F‐EVs.

In general, this study innovatively constructed a 4D@F‐EVs composite delivery system by loading FGF2‐modified engineered EVs (F‐EVs) onto a 4D‐printed chitosan‐based thermosensitive hydrogel (4D‐CTH). By virtue of 4D printing, the scaffold features uniform pore size and regulable morphology, which affords a stable microenvironment and long‐term retention for EVs, along with excellent antibacterial activity and biocompatibility, thus effectively overcoming the technical bottlenecks of conventional FGF2 including low local delivery efficiency and short action duration. Multi‐dimensional investigations including in vitro cell experiments, large animal infected burn model validation and single‐cell RNA sequencing (scRNA‐seq) demonstrated that the 4D@F‐EVs system not only promotes Fb proliferation similar to conventional FGF2 hydrogels, but also induces Fb to secrete KGF and enhance paracrine crosstalk with KC, thus stimulating KC proliferation and facilitating the interaction between Fb and KC, which coordinately modulates wound repair and ultimately achieves efficient regenerative repair of infected burn wounds (Figure 1).

## 2 Materials and Methods

### 2.1 Materials

Chitosan (CTS, deacetylation degree ≥95%, viscosity 100–200 mPa·s) was purchased from Macklin (Shanghai, China). Carboxymethyl chitosan (CMCTS, deacetylation degree of 96.44%, molecular weight 134 kDa) and β‐glycerophosphoric (β‐GP) acid disodium were purchased from Solarbio (Shanghai, China). Stem cell special medium, EVs‐depleted fetal bovine serum and serum replacement were provided by Dakewe (Shenzhen, China); adipogenic, osteogenic and chondrogenic induction kits were purchased from OriCellbio (Guangzhou, China). DMEM/F12 medium, RPMI 1640 medium, high‐glucose DMEM medium, phosphate‐buffered saline (PBS), and fetal bovine serum (FBS) were provided by Meilunbio (Dalian, China). Trypsin and penicillin‐streptomycin were purchased from Solarbio (Shanghai, China). Flow cytometry characterization kit was purchased from BD (Franklin Lakes, NJ, USA). H&E staining, Mmasson and Sirius Red staining kits were provided by Solarbio (Shanghai, China). Flow apoptosis kit and Calcein‐AM/PI live/dead cell staining solution were obtained from Meilunbio (Dalian, China). Unless otherwise stated, all reagents were of the highest commercially available grade and could be used without further purification.

### 2.2 Cells and Animals

Human immortalized epidermal cells (HaCaT cells) and human skin fibroblasts (HSF cells) were purchased from OriCellbio (Guangzhou, China). Human adipose‐derived mesenchymal stem cells (ADMSCs) were isolated from patients following liposuction at the Department of Plastic and Reconstructive Surgery, Qingdao Affiliated Hospital. The patient serum and tissue samples were obtained from the Clinical Laboratory and the Department of Burn and Plastic Surgery, Qingdao Central Hospital. All human‐related experimental protocols were approved by the Ethics Committee of Qingdao Medical College, Qingdao University (Ethical Approval No. QDU‐HEC‐2023246). Rongchang pigs (8 weeks old, 20–30 kg, equal number of males and females) were purchased from the Sichuan Institute of Laboratory Animal Research (Chengdu, China). All animal experiments were approved by the Animal Ethics Committee of the Sichuan Institute of Animal Research (Ethical Approval No. 2024‐002).

### 2.3 Extraction and Characterization of human ADMSCs

#### 2.3.1 Extraction of ADMSCs

The minced adipose tissue was digested with 0.1% collagenase, shaken on a shaker at 150 rpm for 60 min, and centrifuged at 600 × g for 10 min at 22°C. The supernatant was then discarded, and the precipitate was washed with 0.9% normal saline and centrifuged again for 10 min. The cell pellet was harvested and seeded into cell culture flasks for subsequent culture.

#### 2.3.2 Differentiation of ADMSCs

According to the instructions of the Adipose‐derived mesenchymal stem cells Osteogenic differentiation kit, ADMSCs were induced to undergo osteogenic differentiation. After 24 d, the cells were stained with alizarin red working solution at room temperature for 15 min and observed under a light microscope for osteogenic staining. Similarly, following the instructions of the Adipose‐derived mesenchymal stem cells adipogenic differentiation kit, ADMSCs were induced to undergo adipogenic differentiation. After 26 d, the cells were stained with Oil Red O working solution at room temperature for 30 min and observed under a light microscope for adipogenic staining. Similarly, following the instructions of the Adipose‐derived mesenchymal stem cells chondrogenic differentiation kit, ADMSCs were induced to undergo chondrogenic differentiation. After 24 d, the chondrocytes were embedded, sectioned, and stained with Alcian Blue working solution at room temperature for 1 h. Finally, panoramic scanner (3DHISTECH Pannoramic, Hungary) was used to observe the chondrocyte staining effect.

#### 2.3.3 Identification of ADMSCs

Positive markers CD90, CD105, CD73, mixed negative markers (CD34, CD11b, CD19, CD45, HLA‐DR), and isotype controls (mIgG 1, κ, mIgG 2a, κ, mIgG 2b, κ) were applied to characterize ADMSCs. The cells were incubated with solution containing 5 µL of each aforementioned antibody at 4°C in the dark for 30 min, then washed twice with cell staining buffer. The cells from each group were resuspended in 200 µL of staining buffer and analyzed on a flow cytometer (Beckman Coulter, Inc.) for detection of each surface marker.

### 2.4 Production and Characterization of 4D@F‐EVs

#### 2.4.1 Isolation and Preparation of ADMSCs‐derived EVs

ADMSCs were cultured in complete medium containing EVs‐depleted serum. The supernatant of cell culture medium during P3‐P6 phase were centrifuged twice at 4°C (3,000 ×g, 10 min). Between the two centrifugations, the supernatant were filtered through a 0.22 µm filter and collected. The centrifuged supernatant were transferred to ultrafiltration tube (Millipore) and centrifuged at 4°C (3,500 ×g, 30 min). The supernatants obtained after ultrafiltration were transferred to conventional centrifuge tubes and were sequentially centrifuged at 4°C (2,000 × g, 10 min) and at 4°C (10,000 × g, 30 min). The resultant liquid was loaded into ultracentrifuge tubes, and ultracentrifugation was performed at 4°C (120,000 × g, 70 min) on an ultracentrifuge (Eppendorf SE, Germany), after which purified EVs were harvested.

#### 2.4.2 Preparation of PDL‐Modified and FGF2‐Loaded EVs (F‐EVs)

500 µL of EVs solution with a concentration of 7.8×10^10^·particles·mL^−1^ was mixed with 500 µL of 5 mg·mL^−1^ poly‐D‐lysine (PDL, molecular weight: 30–70 kDa, Meilunbio, China) solution. The mixture was gently shaken and incubated at 4°C for 30 min to allow PDL to bind to the EVs surface through electrostatic adsorption. Subsequently, the reaction solution was transferred to a 100 kDa ultrafiltration centrifuge tube (Millipore, USA) and centrifuged at 4,000 × g for 20 min at 4°C; the filtrate was discarded, and the retentate was washed three times with PBS to remove free PDL. The retentate was resuspended in 20 mM Tris‐HCl buffer (pH 7.4) and loaded onto a pre‐equilibrated cation‐exchange chromatography column (HiTrap SP HP, Cytiva). After extensive washing to remove unmodified EVs, the bound PDL‐EVs were eluted, and the elution peak was collected.

The eluent was concentrated using a 100 kDa ultrafiltration tube, and the buffer was exchanged to PBS to yield purified PDL‐EVs. The purified PDL‐EVs were incubated with 1 mL of 2 µg·mL^−1^ FGF2 (17 kDa, Proteintech, China) under gentle shaking at room temperature for 30 min to facilitate FGF2 binding to the PDL‐EVs surface. The mixture was then transferred to a new 100 kDa ultrafiltration tube and centrifuged at 4,000 × g for 20 min at 4°C. The retentate was washed three times with PBS to thoroughly remove unbound FGF2, and the final retentate was collected as purified F‐EVs. The product was aliquoted and stored at ‐80°C until further use.

#### 2.4.3 Preparation of Thermosensitive Hydrogel and 4D Printed Scaffolds

0.1‐0.3 g CTS was dissolved in 4.5 mL of 0.1 mM acetic acid solution, stirred for 10 h, and then mixed with 2.2‐6.7% CMCTS and 6–8% sodium β‐GP to obtain the chitosan‐based thermosensitive hydrogel (CTH) solution [24]. Then, 500 µL of F‐EVs solution (0.5 µg·µL^−1^) was added to 10 mL of CTH solution to obtain CTH@F‐EVs. A 4D printer (BioarchitectSR, Regenovo, China) was used to print CTH@F‐EVs into a homogeneous layer‐by‐layer structure with a diameter of 4 cm and a pore size of 200 µm, thereby obtaining 4D@F‐EVs. The specific preparation condition: temperature: 0°C–4°C; air pressure: 0.15 mPa; speed: 0.2 mm·s^−1^. During the preparation and printing process of 4D‐CTH, 0.5 mg·mL^−1^ of Rhodamine B was added to the CTH solution to achieve a clearer presentation of the gel structure.

#### 2.4.4 Comprehensive Characterization of 4D@F‐EVs

A rheometer was used to measure the elastic modulus and viscosity of CTH. CTH (printed into sheets with a diameter of 10 mm and a thickness of 1 mm) was placed on a 25 mm diameter support plate of the rheometer, with a gap of 500 µm. Under conditions of a shear frequency of 0.1–10 Hz and a strain of 1%, the storage modulus and loss modulus were measured. Viscosity was measured at temperatures ranging from 0 to 40°C. A Nicolet iS50 FTIR spectrometer (Thermo Scientific, Waltham, MA, USA) was used to record the ATR‐FTIR spectra of CTH samples after freezing and freeze‐drying, with a frequency range of 400 to 4,000·cm^−1^. A scanning electron microscope (Carl Zeiss, Germany) was used to characterize the microstructure of the hydrogel. First, CTH/4D‐CTH/4D@F‐EVs samples were rapidly frozen in liquid nitrogen, quickly fractured, and freeze‐dried to sublimate residual water. Then, the fracture surfaces were subjected to gold sputtering, and SEM micrographs were acquired.

### 2.5 In Vitro Analysis of the Uptake and Release of F‐EVs

#### 2.5.1 In Vitro Analysis of the Uptake of F‐EVs

Labeling working solution was prepared by mixing PKH26 linker and Diluent C (Umibio, China) at a volume ratio of 1:9 in the dark. The working solution was added to EVs suspension and incubated for 10 min, followed by centrifugation of the mixture at 120,000 × g for 90 min. HSF cells which had been starved beforehand for 24 h, were inoculated into sterile cell crawls of 24‐well plates. PKH26‐labeled EVs were added and incubated for a total of 12 h. Cell precipitates were rinsed with PBS, fixed with 4% paraformaldehyde for 15 min, and finally washed twice with PBS. Cytoskeleton were stained with phalloidin and the nucleus were counterstained with DAPI (Servicebio, China). Fluorescent images showing intracellular uptake of F‐EVs were captured on a laser scanning confocal microscope (Olympus, Japan).

#### 2.5.2 In Vitro Analysis of the Release of F‐EVs

4D@F‐EVs hydrogel discs with 4 cm diameter were immersed in 10 mL PBS and incubated at 37°C with continuous shaking. At 24, 48 and 72 h post‐incubation, 1 mL of the supernatant was collected at each time point. The BCA protein concentration assay was adopted to measure 4D@F‐EVs. OD values of the solution taken were measured by Microplate Reader (Bio‐Rad, UK), and the release ratio was calculated accordingly.

### 2.6 In Vitro Repair Evaluation of Thermal Stimulation Cells by F‐EVs‐loaded 4D‐CTH

#### 2.6.1 Thermal Stimulation Cells Construction

HaCaT and HSF cells in the logarithmic growth phase were seeded at a density of 5×10^5^ cells·mL^−1^ in a 24‐well plate. After the cells adhered, they were incubated at 54°C for 30 s to obtain thermal stimulation cell.

Co‐culture system construction: 5×10^5^ concentration of thermal‐stimulated HSF cell suspension was added to the upper layer of the Transwell chamber (Corning), and 5×10^5^ concentration of thermal‐stimulated HaCaT cell suspension was added to the lower layer of the Transwell chamber. 1 mL DMEM high glucose culture medium containing 10% FBS or 1 mL extracts of different groups of materials were added to each well and incubated at 37°C with 5% CO_2_.

#### 2.6.2 Groups and Material Process

Materials were immersed in 15 mL high‐glucose DMEM culture medium containing 10% FBS to prepare the extraction solution. UM‐EVs group: 250 µL of EVs (0.5 µg·µL^−1^); FGF2+EVs group: 250 µL of EVs (0.5 µg·µL^−1^) and 250 µL of FGF2 factor (2 µg·mL^−1^); F‐EVs group: 500 µL of EVs (0.5 µg·µL^−1^). 4D@F‐EVs group: One 4D@F‐EVs scaffold with a diameter of 4 cm and 500 µL of F‐EVs (0.5 µg·µL^−1^). Subsequently, cell viability and apoptosis were detected by CCK‐8, live/dead cell staining, and flow cytometry (Supplementary File S1).

### 2.7 In Vivo Therapeutic Evaluation of Burn Wounds by F‐EVs‐loaded 4D‐CTH

Experimental animals were anesthetized using inhaled isoflurane (induction concentration 3–5%) and maintained under continuous inhalation anesthesia during surgery. Preoperative sedation was achieved via intramuscular injection of Zoletil 50 (Tiletamine/Zolazepam, 4 mg/kg). For wound management, after burn modeling, the wound was cleaned daily with saline. Following 3 days of debridement, interventions were implemented according to the respective experimental protocols: In the 4D@F‐ EVs group, the hydrogel dressing was directly applied to the wound and securely fixed; in the other drug‐administered groups, the medication was evenly applied to the wound surface and covered with sterile gauze. All animals were housed individually postoperatively, with gauze changed twice weekly until the experimental endpoint.

To accurately mimic the pathological features of typical clinical wounds, a deep second‐degree infected burn model was established using SPF Rongchang pigs. The diameter of each wound was set to 4 cm, and high consistency between the pathological manifestations of the animal model and clinical patient lesions was guaranteed. Specifically, healthy Rongchang pigs (n = 3) were selected as experimental subjects. Prior to the experiment, the dorsal skin was prepared and routinely disinfected. A self‐developed constant‐temperature burn device (PCT invention patent No.2025/00304) was used to create five burn wounds on the back of each pig, comprising five experimental groups: Ctrl, FGF2, FGF2+EVs, F‐EVs, and 4D@F‐EVs. Inter‐wound distances were strictly maintained at > 4 cm. Burn parameters strictly adhered to the patented method: device temperature, contact pressure, and contact time were set at 150°C, 30 N, and 30 s, respectively (Figure S1), ultimately forming the 4 cm diameter circular deep second‐degree burn wound on the back. Following wound preparation, A suspension of *Staphylococcus aureus* (*S. aureus)* (200 µL, 1 ×10 ^7^ CFU·mL^−1^) was added into the wounds and uniformly applied to the wound surface to simulate the infected microenvironment of clinical burn wounds. The wound was then securely covered and fixed with sterile dressings, successfully establishing a deep second‐degree burn model exhibiting typical infection characteristics.

### 2.8 Single‐Cell Sequencing Analysis

Single‐cell sequencing(scRNA‐seq) samples were obtained from 3 pigs each in the 4D@F‐EVs group and the Control group. Skin tissue was collected from the central area of the burn wound on the back of each pig 35 days after burn modeling. Then, sequencing samples were shipped to LC‐Bio Technology Co., Ltd. at −80°C for scRNA‐seq.

Illumina's bcl2fastq software (version 2.20) was used to demultiplex the sequencing results, which was then converted to the FASTQ format. The Cell Ranger process (version 6.1.1, see 10× Genomics Support for more information) was used for sample demultiplexing, barcode processing, and single‐cell 3' gene counting. scRNA‐seq data were aligned to the Ensembl Genome *Sus scrofa* reference genome. Subsequently, Cell Ranger output was imported into Seurat (version 3.1.1). Low‐quality cells with fewer than 500 genes, or more than 25% mitochondrial genes were excluded. The remaining high‐quality cell data were then used for subsequent advanced bioinformatics analysis.

### 2.9 Bioinformatics Analysis

Bioinformatics analyses subsequent to scRNA‐seq included cell clustering, visualization and annotation, pseudo‐temporal analyses, cell communication analyses, and functional enrichment analyses. The Seurat package (version 4.1.1) was used for cell clustering and sub‐clustering, and UMAP was used for dimensionality reduction. FDR < 0.05 and |log2 Fold Change| > 0.26 were considered to be significantly different. The ClusterProfiler package in R was used to perform KEGG and GO analyses. The relevant conclusions were further verified by the corresponding WB analysis (Supplementary File S1, Supplementary Table S1).

For molecular docking, X‐ray structure of FGF2 from the PDB database: 8OM6 (resolution: 1.31 Å), The 3D structure of PDL in SDF format was obtained from the PubChem database (CAS: 27964‐99‐4), KGF full‐length AlphaFold predicted structure (Uniprot ID: P21781), KGFR (Uniprot ID: P21802) X‐ray structure 6V6Q was obtained from Uniprot database. HDOCK and AutoDock Vina 1.2.2 were used for molecular docking analysis. PLIP and LigPlus binding interfaces for protein‐ligand complexes were used to perform systematic analyses, and PyMOL 2.5 software was used to complement the interaction‐related details. The local HDOCKlite v1.1 server was used for protein‐protein docking.

### 2.10 Statistical Analysis

GraphPad Prism 9.0 software (GraphPad software, Inc., USA) was used for the statistical analysis. All quantitative data were expressed as mean ± standard deviation, and one‐way ANOVA was used for comparison between groups. *p* < 0.05 was considered statistically different (*P < 0.05; **P < 0.01; ***P < 0.001; ****P < 0.0001).

## 3 Results

### 3.1 Preparation and Characterization of F‐EVs

**FIGURE 2:**
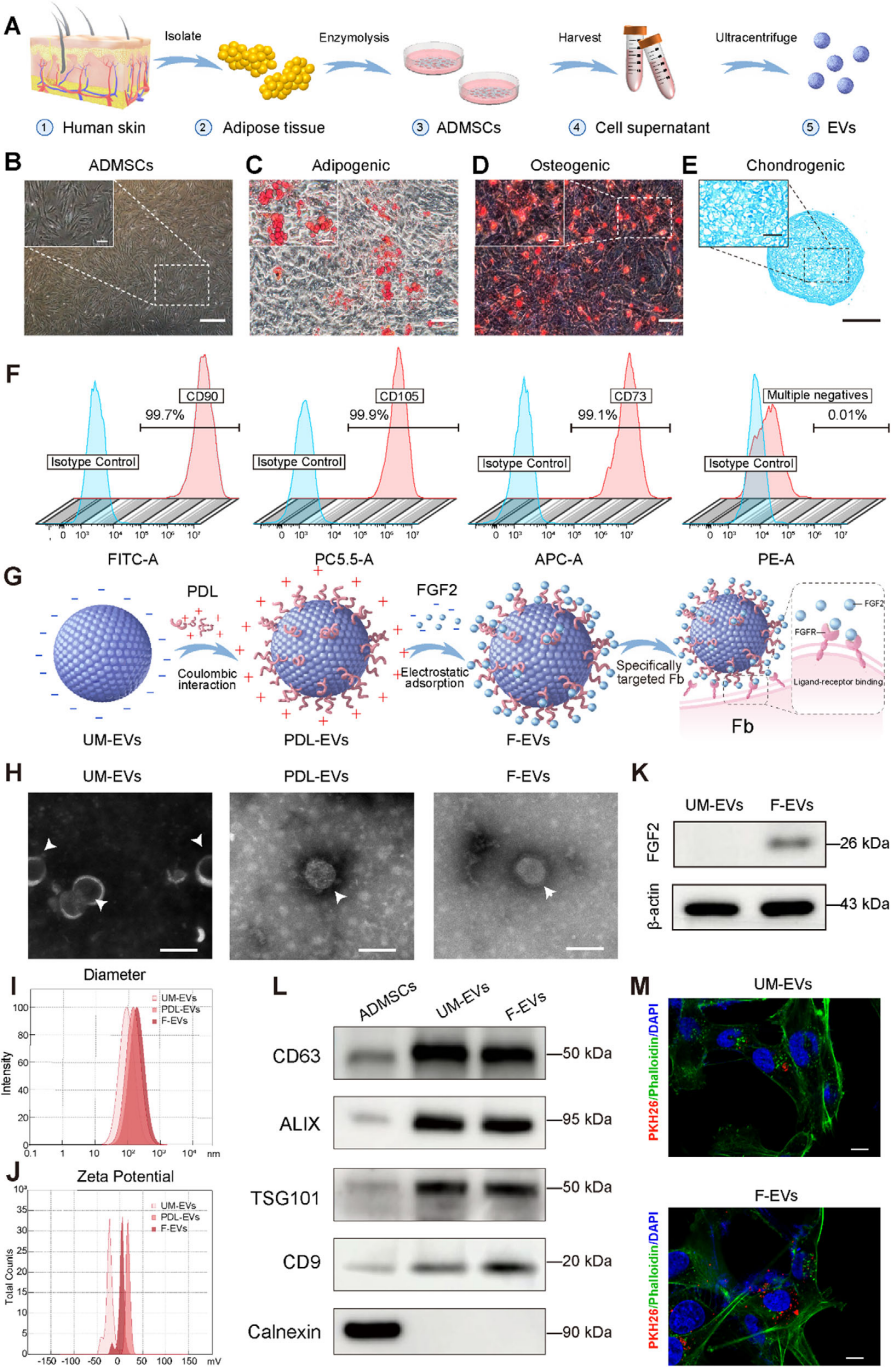
F‐EVs Preparation and identification. (A) Acquisition diagram of ADMSCs and EVs. Subcutaneous adipose tissue was obtained from patients undergoing liposuction and subsequently used for isolation to obtain more purified fat. After digestion with type I collagenase, ADMSCs were harvested. During the culture process, the supernatant was collected and used for EVs extraction. ADMSCs, adipose‐derived mesenchymal stem cells; Extracellular Vesicles, EVs. (B) Representative images of P3 ADMSCs; Scale bar: 500 µm (bottom), 100 µm (up). (C‐E) Representative images of (C) adipogenic, (D) osteogenic; Scale bar: 200 µm (bottom), 50 µm (up). (E) chondrogenic differentiated ADMSCs; Scale bar: 100 µm (bottom), 25 µm (up). (F) Expression of ADMSCs surface markers CD90, CD105, CD73, mixed negative markers (CD34, CD11b, CD19, CD45, HLA‐DR) and homologous controls (mIGg 1, κ, mIGg 2a, κ, mIGg2b, κ) were analyzed by flow cytometry. (G) Engineered EVs preparation schematic diagram. PDL, poly‐D‐lysine; FGF2, fibroblast growth factor 2; UM‐EVs, unmodified EVs; PDL‐EVs, PDL modified EVs; F‐EVs, FGF2 modified EVs by PDL. (H) TEM images of EVs, Scale bar: 100 nm. (I) DLS to detect the particle size distribution of EVs. (J) Zeta potential distribution of EVs. (K) FGF2 protein were detected by WB. (L) EVs‐specific marker proteins CD63, ALIX, TSG101, CD9 and negative protein Calnexin was detected by WB. (M) Uptake of UM‐EVs and F‐EVs by Fb. EVs were fluorescently labeled with PKH67 (red); Cytoskeleton were stained with phalloidin (green); Cell nuclei were stained with DAPI (blue); Sacle bar: 10 µm.

In order to prepare F‐EVs, primary human adipose mesenchymal stem cells (ADMSCs) and corresponding EVs were first successfully extracted and identified (Figure 2A). Oil red O, alizarin red, and alcian blue staining were performed on spindle‐shaped ADMSCs (Figure 2B), confirming that ADMSCs possessed satisfactory adipogenic, osteogenic, and chondrogenic differentiation capacities (Figure 2C–E). In addition, flow cytometry analysis showed high expression of ADMSC‐positive markers (CD90: 98.20 ± 1.95%, CD105: 98.93 ± 1.42%; CD73: 97.87 ± 2.58%) and low expression of negative markers CD34, CD11b, CD19, CD45 and HLA‐DR (0.13 ± 0.19%), which verified the high purity of ADMSCs (Figure 2F) and facilitated the acquisition of high‐purity ADMSC‐derived EVs. Further, unmodified EVs (UM‐EVs) derived from ADMSC were first grafted with PDL to obtain PDL‐modified EVs (PDL‐EVs), which were then prepared into FGF2‐modified EVs (F‐EVs) by electrostatic adsorption (Figure 2G). The concentration of raw UM‐EVs for constructing F‐EVs was measured to be 7.8×10^10^ particles·mL^−1^ by nanoparticle tracking analysis (NTA) (Figure S2A). The surface charge changes of PDL‐EVs complexes at different volume ratios were investigated through Zeta potential analysis. The results showed that the surface potential of the complexes increased gradually with the rise in PDL proportion. A volume ratio of 1:1 enabled effective positive charge modification on the EVs surface and achieved charge saturation at a low dosage, laying a solid foundation for the subsequent stable electrostatic binding of FGF2 (Figure S2B). Transmission electron microscopy (TEM) was used to characterize the morphologies of unmodified EVs (UM‐EVs), poly‐D‐lysine (PDL)‐modified EVs (PDL‐EVs), and FGF2‐modified EVs (F‐EVs), confirming that UM‐EVs exhibited the typical cup‐shaped morphology, while PDL‐EVs and F‐EVs showed corresponding morphological characteristics after polymer modification (Figure 2H). Dynamic light scattering (DLS) provided particle sizes of UM‐EVs (98.35±9.36 nm), PDL‐EVs (141.53±5.74 nm) and F‐EVs (191.51±11.59 nm) (Figure 2I), which were consistent with TEM results. During the preparation of UM‐EVs from intermediate PDL‐EVS to F‐EVs, the zeta potential changed from ‐15.90±2.71 mA to 18.53±0.64 and finally 4.33±0.81 mA (Figure 2J), which further verified the successful preparation of F‐EVs. Molecular docking analysis further confirmed that in addition to the electrostatic adsorption of FGF2, PDL can also form hydrogen bonds with LYS – 86, TYR – 133, VAL – 49 residues of FGF2, a salt bridge with LYS – 86, and hydrophobic interactions with ARG‐48 (Figure S3). These multiple interactions contribute to the stable construction of F‐EVs and facilitate the targeted delivery of FGF2. Western blotting analysis showed that the expression level of FGF2 in F‐EVs was significantly higher than that in UM‐EVs (Figure 2K; Figure S4A), confirming the successful modification of EVs with FGF2.

In addition, significantly higher expression of positive markers (CD63, TSG101, Alix) of F‐EVs and UM‐EVs than ADMSCs and the undetected negative marker (Calnexin) of EVs (Figure 2L), demonstrated uninfluenced EVs properties after FGF2 modification. Laser confocal images of PKH26 (red fluorescent probe) (Figure 2M; Figure S4B) confirmed higher Fb uptake of F‐EVs than UM‐EVs, indicating the promotion of FGF2 modification on EVs delivery targeting Fb. To further clarify the targeting mechanism of F‐EVs at the molecular level, additional Co‐IP assays were performed to directly verify the specific interaction between FGF2 and FGFR1 on the target cell surface. FGFR1 antibody was used for protein pulldown, and co‐precipitated FGF2 was detected. Results directly confirmed that F‐EVs achieve targeted delivery through specific binding of surface‐modified FGF2 to FGFR1 on target cells (Figure S5). This specific binding, on one hand, promotes the enrichment of engineered EVs around Fb; on the other hand, it drives their uptake by Fb through endocytosis, thereby achieving the targeted presentation of FGF2. In summary, the engineered F‐EVs targeting Fb was successfully prepared after ADMSCs and EVs with high purity were obtained.

### 3.2 Preparation, Characterization and Antibacterial Performance Evaluation of 4D@F‐EVs

**FIGURE 3:**
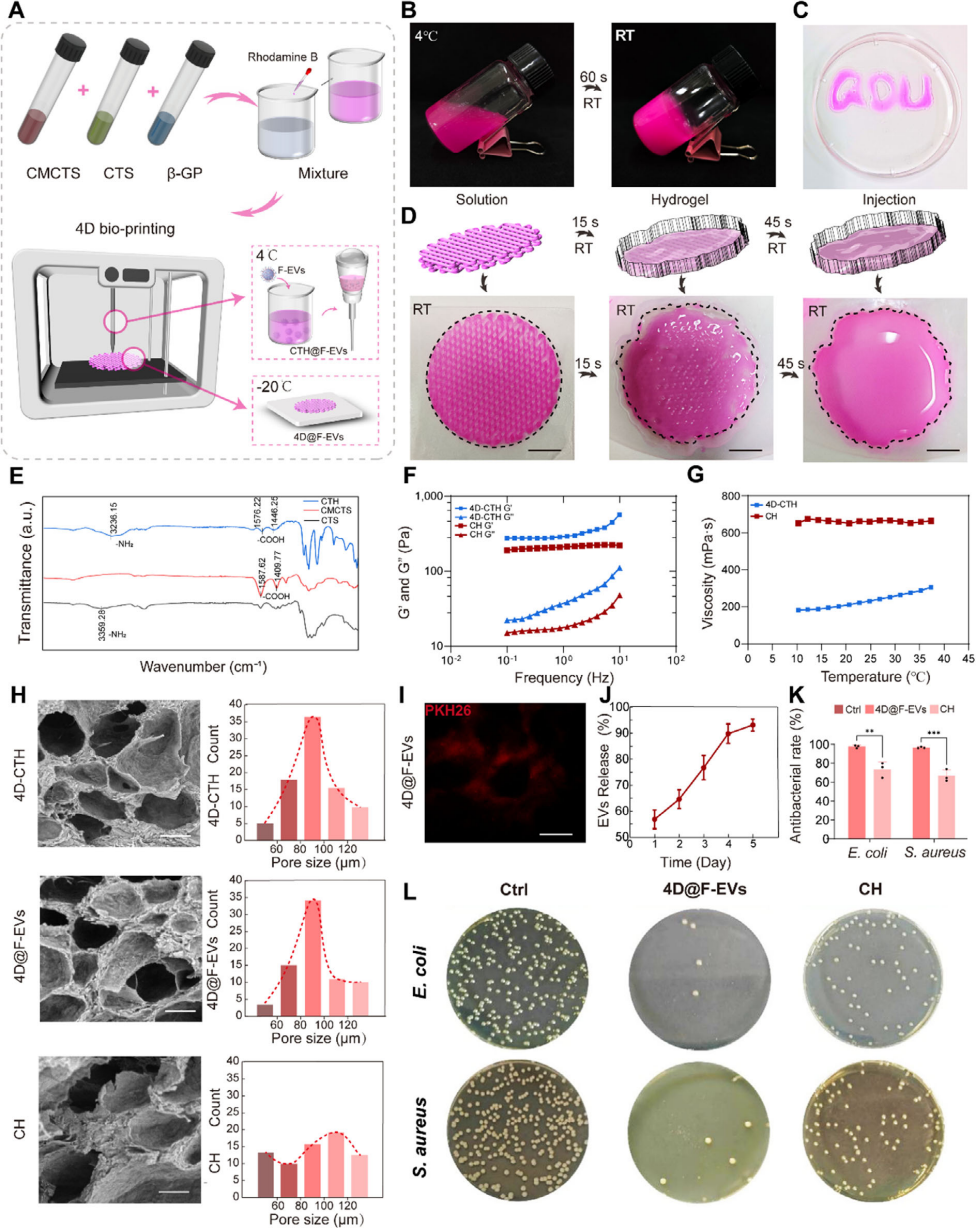
Fabrication, characterization and antibacterial assessment of the 4D@F‐EVs. (A) Fabrication process of the 4D bioprinting bioink. The CMCTS, CTS, β‐GP and a dye were uniformly mixed before being placed in a 4D bioprinter to print at 4°C and −20°C, yielding two EVs carriers (CTH@F‐EVs and 4D@F‐EVs). Chitosan: CTS; carboxymethyl chitosan: CMCTS; β‐glycerophosphate sodium: β‐GP. (B) The thermosensitive CTH bioink transitions from a solution to a hydrogel within 60 s at 37°C. (C) The extrudability of CTH bioink. (D) Morphological changes of 4D‐CTH at room temperature (RT) at 15 and 60 s. (E) FTIR analysis for the molecular structure of the samples. (F) Measurement of the storage modulus (G') and loss modulus (G'') vs. frequency. (G) Measurement of the viscosity vs. temperature. (H) Observation of the micro—morphologies of CH, 4D‐CTH and 4D@F‐EVs and analysis of the pore—diameter distribution by SEM. Scale bar: 100 µm. (I) Laser confocal observation of PKH26‐labeled F‐EVs in 4D‐CTH. Scale bar: 100 µm. (J) Quantitative determination of BCA protein for the analysis of EVs release behavior. (K) Antimicrobial effects of different groups against *E. coli* and *S. aureus*. Scale bar: 1 cm. (L) Quantitative results of the antibacterial activity. *P < 0.05; **P < 0.01; ***P < 0.001; ****P < 0.0001.

The CTS/CMCTS/β‐GP thermosensitive hydrogel (CTH) solution mixed with F‐EVs was subject to 4D bioprinting to prepare 4D@F‐EVs (Figure 3A). The CTH exhibited excellent thermosensitive properties. Specifically, the CTH solution remained in a fluid state at 4°C and converted to a gel state at 37°C (Figure 3B), showing irreversible gelation with increasing temperature. Moreover, the fluid CTH solution at 4°C presented a good injectable state (Figure 3C), which ensured the smooth implementation of 4D printing. The 4D‐CTH obtained after 4D printing exhibits regular shape and uniform pores. Meanwhile, due to the solution‐to‐gel transformation, it exhibited excellent deformability, enabling it to adapt to irregular wound shapes (Figure 3D), thereby achieving a closer fit with the wound. The Fourier‐transform infrared spectroscopy (FTIR) revealed the molecular structural characteristics of CTH, which had the characteristic absorption peaks of carboxymethyl chitosan ‐COO^−^ asymmetric and symmetrical stretching vibration and the characteristic absorption peaks of chitosan amino‐NH_2_ stretching vibration (Figure 3E). The rheological tests showed that at frequencies ranging from 0.01 to 1000 Hz and within the temperature range of 10 to 40°C, storage modulus (G') of CTH was always greater than loss modulus (G''). Additionally, as the temperature increased, 4D‐CTH exhibited higher viscosity, confirming the temperature‐sensitive property of CTH.

To systematically characterize the performance advantages of 4D‐CTH hydrogel, compared to conventional chitosan hydrogel (CH), while 4D‐CTH showed a significantly higher G' value and better structural stability over the entire testing frequency range, possessing more favorable viscoelasticity and mechanical tolerance adapted to the dynamic physiological environment of burn wounds (Figure 3F,G). The scanning electron microscope (SEM) confirmed that CH, 4D‐CTH, and 4D@F‐EVs all exhibited a network structure. However, the results showed that the average pore size of 4D‐CTH was (95 ± 15) µm, which was slightly smaller than that of CH (110 ± 25) µm, but with a significantly narrower and more uniform pore size distribution (*p < 0.05). Moreover, the pore size of 4D‐CTH was concentrated in the ideal range of 80–100 µm, which was more suitable for cell growth and nutrient exchange compared with the discrete pore size distribution of CH (60‐120 µm) (Figure 3H). After incubating L929 Fb with extracts of 4D‐CTH and CH hydrogels for 24 h, Calcein‐AM/PI live/dead staining showed that the cell viability of the 4D‐CTH group was significantly higher than that of the CH group and slightly higher than that of the Ctrl group, confirming that 4D‐CTH was non‐cytotoxic and exhibited superior biocompatibility and cellular regulatory ability compared with CH (Figure S6A, B). Furthermore, fluorescence analysis confirmed that the PKH26‐labeled F‐EVs were evenly distributed in the 4D‐CTH and maintained good activity (Figure 3I). Additionally, the BCA protein quantification results showed that 4D‐CTH could achieve uniform and continuous release of F‐EVs, and the effective concentration maintained at the wound site for more than 5 days (Figure 3J).

To systematically evaluate the in vitro antibacterial activity of 4D@F‐EVs, a multi‐dimensional approach was adopted in this study to characterize its antibacterial mechanism and endotoxin clearance efficacy. Results of the spread plate assay showed that compared with the control group, the number of colony‐forming units (CFU) in the 4D@F‐EVs treatment group was significantly reduced with a markedly elevated antibacterial rate (Figure 3K,L). Crystal violet staining confirmed that 4D@F‐EVs could effectively inhibit bacterial biofilm formation, and quantitative analysis of the crystal violet OD value via a microplate reader indicated a significant decrease in the total amount of biofilms (Figure S7A,B). DMAO green fluorescence was used to label the total number of bacteria, while propidium iodide red fluorescence specifically labeled dead bacteria with impaired membrane integrity; fluorescence quantitative analysis revealed a significant increase in the proportion of dead bacteria, demonstrating that 4D@F‐EVs exerts a bactericidal effect by disrupting the bacterial membrane structure (Figure S7C,D). SEM observation directly exhibited morphological changes on the bacterial surface, including cell shrinkage, membrane rupture and cytoplasmic leakage, which confirmed its membrane damage mechanism from a morphological perspective (Figure S7E). In addition, the endotoxin clearance assay demonstrated that 4D@F‐EVs possessed high‐efficiency endotoxin removal capacity (Figure S7F). In summary, 4D@F‐EVs exerts its antibacterial effects by directly disrupting the integrity of bacterial cell membranes to induce bacterial death, inhibiting biofilm formation to reduce the risk of persistent infection, and reducing endotoxin levels.

To enhance the clinical utility of 4D@F‐EVs, we systematically investigated its storage conditions and stability. Results indicate that 4D@F‐EVs maintain an intact and relatively uniform pore structure after 3 months of storage at −80°C and within 4 h at room temperature (Figure S8A). Following 3 months of storage at −80°C, samples rapidly respond to temperature changes at room temperature, forming a skin‐adhering gel state. Dynamic rheology testing revealed typical viscoelastic gel behavior, with the storage modulus G' consistently exceeding the loss modulus G''. This viscoelastic characteristic remained unchanged during the 4 h room‐temperature period, fully meeting intraoperative handling requirements (Figure S8B). Regarding functional stability, the sustained‐release properties of 4D@F‐EVs showed no significant decline (Figure S8C). Furthermore, NTA revealed that the particle size of F‐EVs remained stable at approximately 100 nm under different storage conditions, with particle concentration maintained within the range of 4.0×10^10^ –4.2×10^10^ particles/mL without significant variation. This indicates no aggregation or rupture, confirming stable physical state (Figure S8D). WB analysis confirmed stable FGF2 content within F‐EVs, with no functional impairment, meeting clinical transport and storage requirements (Figure S8E). In summary, the 4D@F‐EVs constructed in this study offer the possibility for the engineering EVs therapy of burns and scalds by providing an ordered 3D structure, adjustable shape, satisfactory biological safety, antibacterial performance and storage stability.

### 3.3 Direct Promotion of the Proliferation of Thermal Stimulation Fb and Indirect Promotion of the Proliferation of KC by 4D@F‐EVs

**FIGURE 4:**
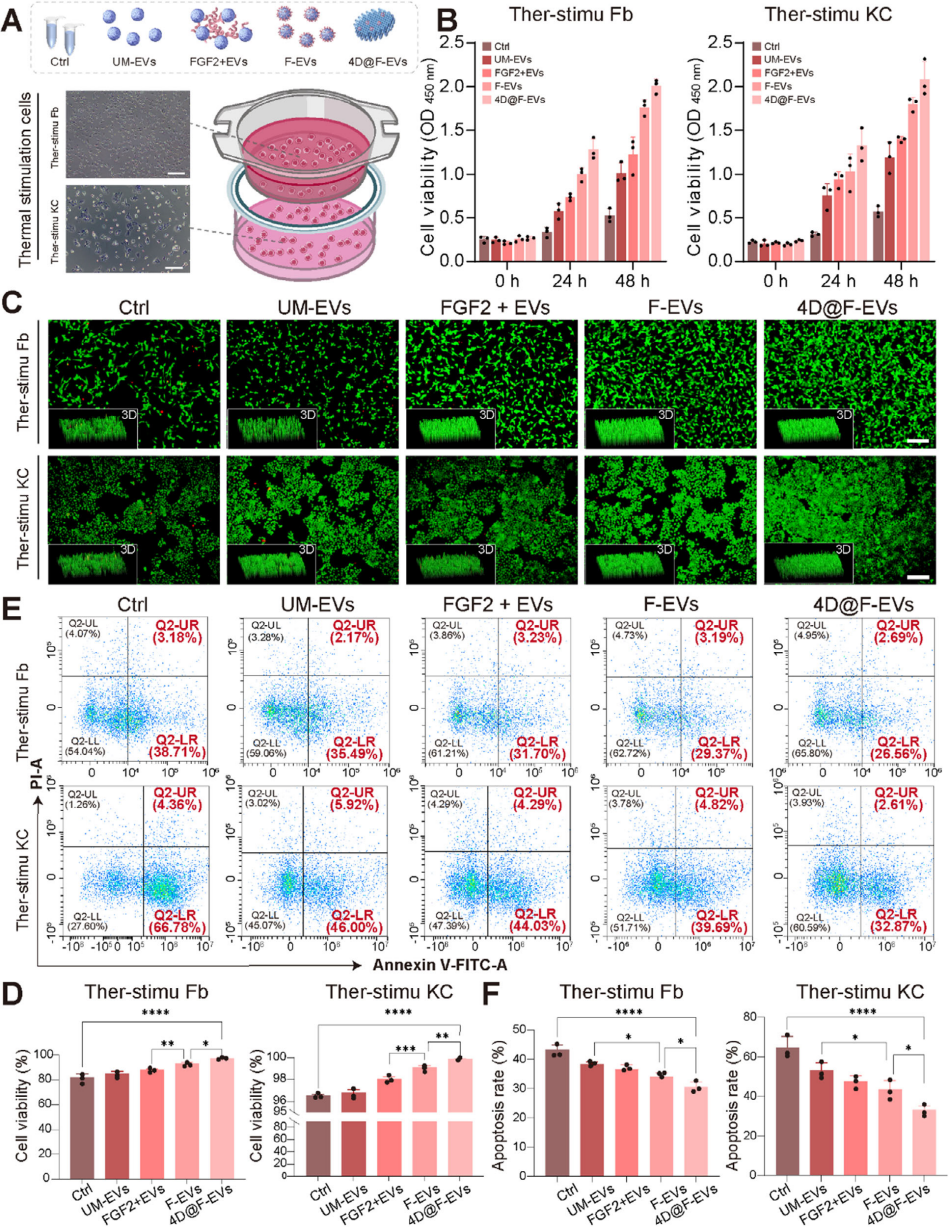
4D@F‐EVs in vitro experiments. (A) Schematic of the coculture system of heat‐stimulated KC and Fb; groups include Ctrl (PBS‐treated), Ther‐stimu (thermal‐stimulated), UM‐EVs (unmodified EVs), FGF2+EVs (FGF2 combined with EVs), F‐EVs (FGF2‐modified EVs), and 4D@F‐EVs (4D scaffold loaded with F‐EVs), Scale bar: 100 µm. (B) CCK8 assay for Fb and KC proliferation (n = 3). (C) Fb and KC live/dead fluorescence images and 3D‐viewed merged fluorescence intensity; green for live cells, red for dead cells, scale bar: 100 µm. (D) Live/dead staining for cell viability analysis (n = 3). (E) Flow cytometry for Fb apoptosis. (F) Flow cytometry—based apoptosis analysis (n = 3). *P < 0.05; **P < 0.01; ***P < 0.001; ****P < 0.0001.

Fb expressing FGFR, the receptor for FGF2 binding, are the core effector cells for dermal repair and remodeling [25, 26, 27]. Meanwhile, KC also plays an important role in epidermal wound repair and closure [28, 29]. To further investigate whether 4D@F‐EVs can directly promote Fb proliferation and indirectly enhance keratinocyte (KC) proliferation, a co‐culture system was established using thermally stimulated Fb and normal KC. Thermally stimulated Fb exhibited significant morphological alterations, including increased cell volume, prominent nucleoli, and reduced intercellular connections, confirming that thermal stimulation exerted a significant adverse effect on the normal physiological functions of skin tissue (Figure 4A). CCK‐8 assay results showed that 4D@F‐EVs treatment could significantly reverse the thermal injury‐induced impairment and enhance the proliferative capacity of both Fb and KC (Figure 4B). Live/dead cell staining confirmed that 4D@F‐EVs treatment notably increased the survival rates of Fb and KC (Figure 4C,D; Figures S9 and S10). Furthermore, flow cytometry analysis demonstrated that the apoptosis rates of Fb and KC were significantly lower in the 4D@F‐EVs treatment group compared with other groups (Figure 4E,F). To exclude potential inherent cytotoxicity of 4D@F‐EVs, AM/PI live/dead cell staining and CCK‐8 cell proliferation assay were used to evaluate its biocompatibility on normal Fb and KC without thermal injury. The results showed no significant difference in cell viability between the 4D@F‐EVs group and the control group. Meanwhile, 4D@F‐EVs exerted an obvious growth‐promoting effect on thermally injured cells (Figure S11A,C). Therefore, the above in vitro results confirmed that 4D@F‐EVs treatment not only could directly intervene in the viability and proliferation ability of Fb after thermal injury, but also could indirectly enhance the viability and proliferation ability of KC after thermal injury, providing an effective strategy for the treatment of burn skin.

### 3.4 4D@F‐EVs Significantly Promotes the Burn Healing of Large Animals

**FIGURE 5:**
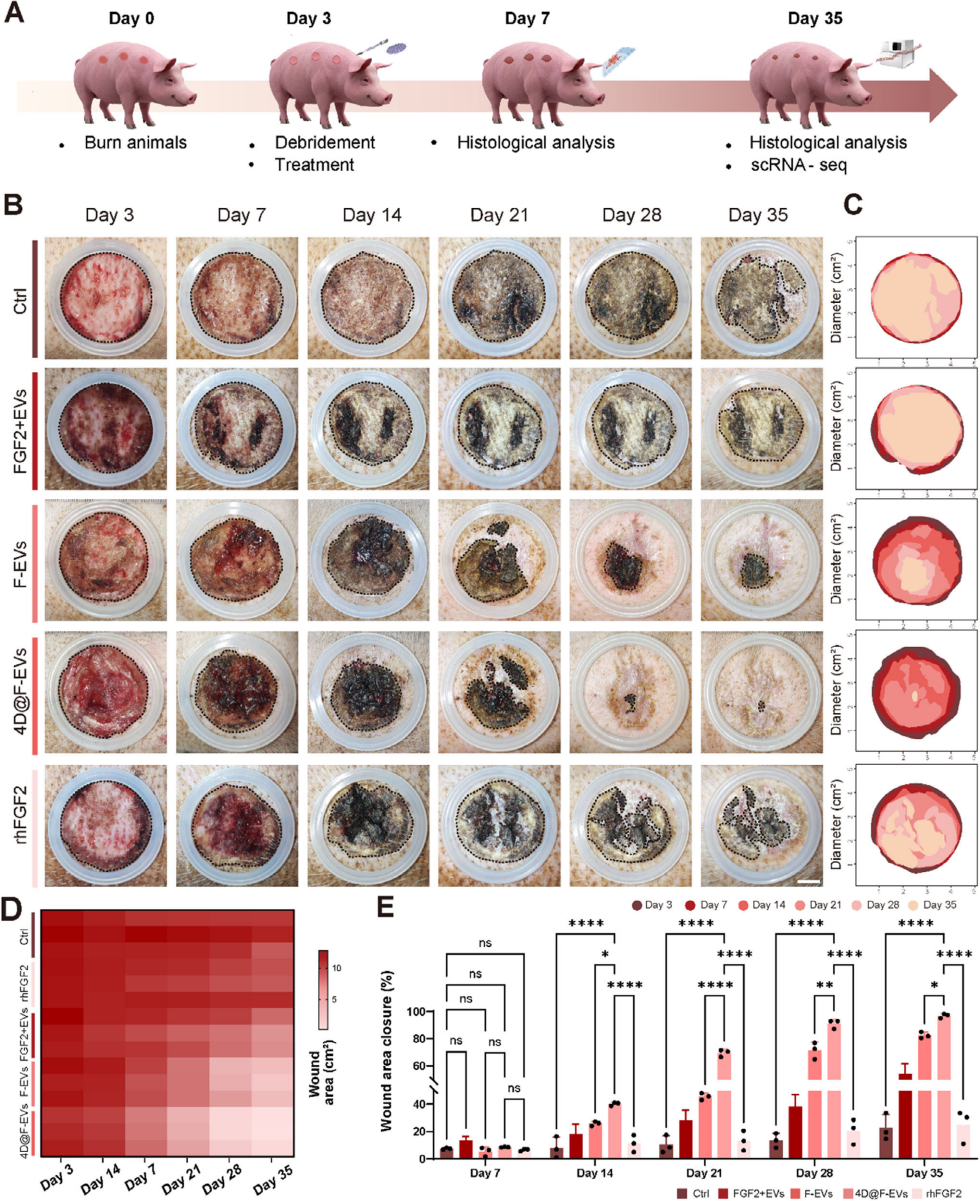
Effects of 4D@F‐EVs on burn infected wound healing in pigs. (A) Schematic diagram of the experimental process. (B) Representative images of wounds in different groups scale bar: 1,000 mm, inner diameter of the gasket 4 cm. (C) Schematic diagram of wound morphology changes over time in different groups. (D) Heat map of wound areas in different groups. (E) Statistical analysis of wound healing rates in different groups. Data are expressed as mean±SD (n = 3), *P < 0.05, **P < 0.01, ***P < 0.001, ****P < 0.0001.

**FIGURE 6:**
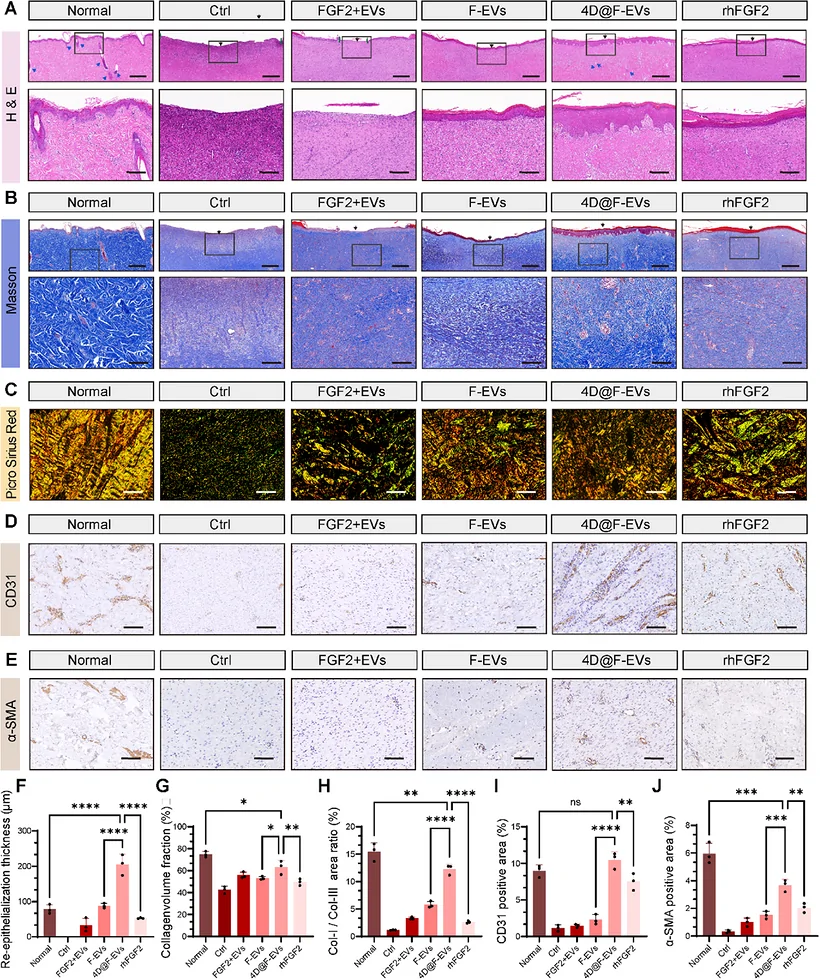
Histological evaluation. (A) H&E staining. Scale bars: 1,000 µm (upper), 200 µm (lower). Black arrows: wound center; blue arrows: new hair follicles; green lines: edges of unhealed wounds. (B) Masson's staining. Scale bars: 1,000 µm (upper), 200 µm (lower). Black arrows: wound center. (C) Picro Sirius Red staining. Scale bar: 200 µm. Immunohistochemical staining of CD31 (D) and α‐SMA (E); Scale bar: 200 µm. (F‐J) Quantitative analysis of each group (n = 3). Collagen volume fraction = blue collagen area / total collagen area. Col‐I: type I collagen (red or yellow); Col‐III: type III collagen (green). *P < 0.05, **P < 0.01, ***P < 0.001, ****P < 0.0001.

Furthermore, a temperature‐adjustable burn device was used to establish deep second‐degree burn wounds with a diameter of 4 cm on the backs of healthy pigs. As shown in Figure S12A, a yellowish‐white eschar was formed on the surface of the burned skin. Hematoxylin‐eosin (H&E) and Masson staining further confirmed that the burn depth extended to the superficial dermis but did not reach the deep dermis or adipose layer (Figure S12B,C), verifying the successful establishment of the deep second‐degree burn model. The treatment process for the burn wounds after successful animal model construction is illustrated in Figure 5A. Based on the observation and calculation of the wound area at different time points after various treatments (Figure 5B,C), the burn wounds treated with 4D@F‐EVs showed significantly accelerated healing compared with the traditional recombinant human basic fibroblast growth factor (rhFGF2) gel treatment group and the normal saline control group. However, the burn wounds treated with FGF2+EVs did not show obvious healing advantages, which might be related to the difficulty in maintaining the activity of FGF2 and EVs on the surface of the burn wound (Figure 5D,E). H&E staining, Masson staining, Sirius Red staining, and immunohistochemical staining of the treated tissues were obtained. First, hematoxylin‐eosin (H&E) staining demonstrated that on day 35, compared with the untreated group, the skin tissue treated with 4D@F‐EVs exhibited more significant re‐epithelialization, a thicker epidermal layer, and a greater number of hair follicles—findings indicating that 4D@F‐EVs treatment promoted scar‐free skin tissue regeneration (Figure 6A,F). Masson staining and Sirius Red staining further confirmed that the burn tissue treated with 4D@F‐EVs contained more newly formed collagen fibers, with a higher proportion of type I collagen relative to type III collagen. These results suggest that 4D@F‐EVs facilitated more effective dermal healing (Figure 6B,C,G,H). Additionally, immunofluorescence staining confirmed more significant expression of α‐SMA and CD31 after 4D@F‐EVs treatment (Figure 6D,E,I,J). The upregulation of α‐SMA is related to the activation of myofibroblasts, which plays a key role in wound contraction and tissue remodeling [30]. The upregulation of CD31 indicates active angiogenesis, which is crucial for the blood supply and nutrient delivery to the wound. Therefore, 4D@F‐EVs treatment can significantly promote the reconstruction of muscle and vascular tissues in burn wounds. H&E staining of the heart, liver, spleen, lung, and kidney confirmed that 4D@F‐EVs treatment did not cause lesions in internal organs, confirming the biocompatibility of 4D@F‐EVs (Figure S13). In conclusion, 4D@F‐EVs treatment was proven to significantly promote the healing of deep second‐degree large animal burn wounds, providing a highly promising clinical strategy for burn treatment.

### 3.5 scRNA‐seq Analysis of Microenvironmental Changes After 4D@F‐EVs Treatment

**FIGURE 7:**
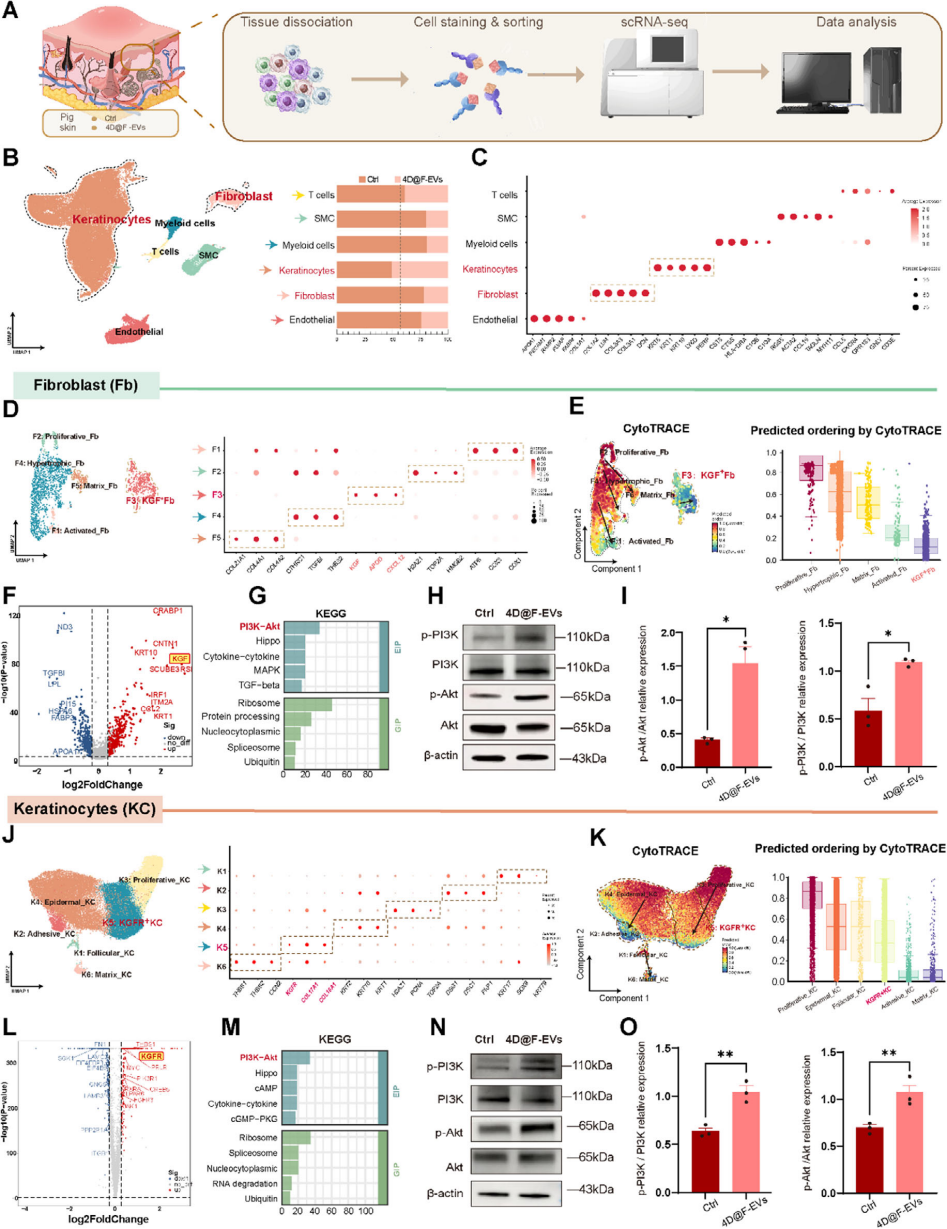
Single‐cell RNA sequencing unveils micro‐environmental alterations following 4D@F‐EVs treatment. (A) Schematic workflow of the sequencing pipeline. (B) UMAP clustering (left) and quantitative comparison of cell numbers between the two groups (right). (C) Dot‐plot depicting signature‐gene expression across distinct cell types. (D) UMAP visualization of Fb subpopulations (left) and their corresponding signature‐gene dot‐plot (right). (E) CytoTRACE pseudotemporal analysis illustrating (left) spatial distribution and (right) inferred differentiation trajectory of Fb subtypes; red denotes early, blue denotes late differentiation. (F, G) Volcano plot (F) and KEGG enrichment (G) of differentially expressed genes in Fb between 4D@F‐EVs and Ctrl. (H) WB validation of p‐PI3K, PI3K, p‐Akt, and Akt in Fb. (I) Quantitative analysis of (H). (J) UMAP visualization of KC subpopulations (left) and their signature‐gene dot‐plot (right). (K) CytoTRACE pseudotemporal analysis showing (left) spatial distribution and (right) inferred differentiation trajectory of KC subtypes; red indicates early, blue indicates late differentiation. (L, M) Volcano plot (L) and KEGG enrichment (M) of differentially expressed genes in KC between 4D@F‐EVs and Ctrl. (N) WB validation of p‐PI3K, PI3K, p‐Akt, and Akt in KC. (O) Quantitative analysis of (N). Data are presented as mean ± SD (n = 3); *p < 0.05, **p < 0.01, ***p < 0.001, ****p < 0.0001.

After 35 days of treatment, skin samples from the 4D@F‐EVs group and the control group were collected for scRNA‐seq. The workflow included tissue dissociation, cell sorting, on‐machine sequencing, and subsequent data analysis (Figure 7A). Through UMAP dimensionality reduction analysis, six major cell types were successfully identified: KC, Fb, endothelial cells, myeloid cells, smooth muscle cells (SMC), and T cells. Among them, the numbers and distributions of KC and Fb in the two groups showed significant differences (Figure 7B; Figure S14). Each cell population exhibited a unique gene expression signature that aligned well with its known biological roles. Specifically, KC highly expressed genes related to the skin barrier function such as *KRT5*, *KRT1*, and *KRT10*, while in Fb, characteristic genes related to Fb proliferation and migration such as *COL1A2*, *COL3A1*, and *DCN* were highly expressed (Figure 7C). Fb, as the main component of the dermis in the skin, were subjected to re‐clustering analysis to further explore the mechanism by which 4D@F‐EVs promote skin dermal healing. First, Fb cells were classified into 5 subgroups through UMAP clustering analysis: proliferative Fb, activated Fb, matrix‐related Fb, KGF^+^Fb, and over‐proliferative Fb. Dot plots were generated to illustrate the gene expression signatures of each subgroup, including the high expression of COL1A1 and COL3A1 in matrix Fb and elevated expression of KGF in KGF^+^ Fb (Figure 7D). Additionally, UMAP clustering analysis of the 4D@F‐EVs group and the control group confirmed the significant differences in the number of these 5 cell subgroups, including a significant reduction in over‐proliferative Fb and a significant increase in KGF^+^Fb, which respectively led to less scar formation and more F‐EVs capture (Figure S15). Time‐series CytoTRACE analysis revealed the spatial distribution and differentiation order of distinct Fb subsets, verifying that KGF^+^ Fb represented a mature differentiation stage. Mature Fb play a key role in the synthesis and secretion of extracellular matrix components such as collagen, which is beneficial for wound filling and the acceleration of healing (Figure 7E). Furthermore, the characteristic expressed genes of Fb were used to analyze the key targets of 4D@F‐EVs in promoting healing. Specifically, it was confirmed that 4D@F‐EVs treatment significantly increased the expression of KGF (FGF7) (Figure 7F; Figure S16). Gene Ontology (GO) and Kyoto Encyclopedia of Genes and Genomes (KEGG) pathway enrichment analysis further highlighted the crucial roles of cell adhesion processes and the PI3K‐Akt signaling pathway in the crosstalk between Fb and KGF^+^Fb (Figure 7G; Figure S17 and S18). Correspondingly, WB further verified the activation of p‐PI3K/PI3K and p‐Akt/Akt signaling pathways in Fb by 4D@F‐EVs (Figure 7H,I).

Re‐clustering analysis of KC at the single‐cell level enabled characterization of subtype dynamics in response to 4D@F‐EVs treatment, thereby facilitating mechanistic exploration of how 4D@F‐EVs accelerate epidermal healing. First, six major KC subtypes were identified, including Follicular_KC, Adhesive_KC, Proliferative_KC, Epithelial_KC, KGFR^+^KC, and Basal_KC. The UMAP clustering graph revealed the distribution of these KC subtypes, and the point graph further showed the expression patterns of specific genes in these KC subtypes, including the high expression of KGFR in KGFR^+^KC (Figure 7J). Additionally, UMAP clustering analysis revealed marked differences in the proportion of these six cell subtypes between the 4D@F‐EVs and control groups. In particular, the proportion of KGF^+^ Fb was significantly elevated, implying that increased KGF expression leads to enhanced activation of KGFR in keratinocytes (Figure S19). The time‐series analysis of CytoTRACE demonstrated the location distribution and differentiation sequence of different KC subtypes (Figure 7K). The analysis of 4D@F‐EVs and the control group in the characteristic expression genes of KC proved significant differences in the expression of genes such as KGFR (Figure 7L, Figure S20). Subsequent GO analysis and KEGG analysis further revealed that 4D@F‐EVs treatment could activate the PI3K‐Akt pathway in KGFR^+^KC (Figure 7 m, Figures S21 and S22), which was further confirmed by WB analysis. Thus, 4D@F‐EVs treatment can further promote KC proliferation and differentiation, as well as damaged skin repair, by activating the PI3K‐Akt signaling pathway in KC (Figure 7N,O).

### 3.6 4D@F‐EVs Promote the Crosstalk Between Fb and KC

**FIGURE 8:**
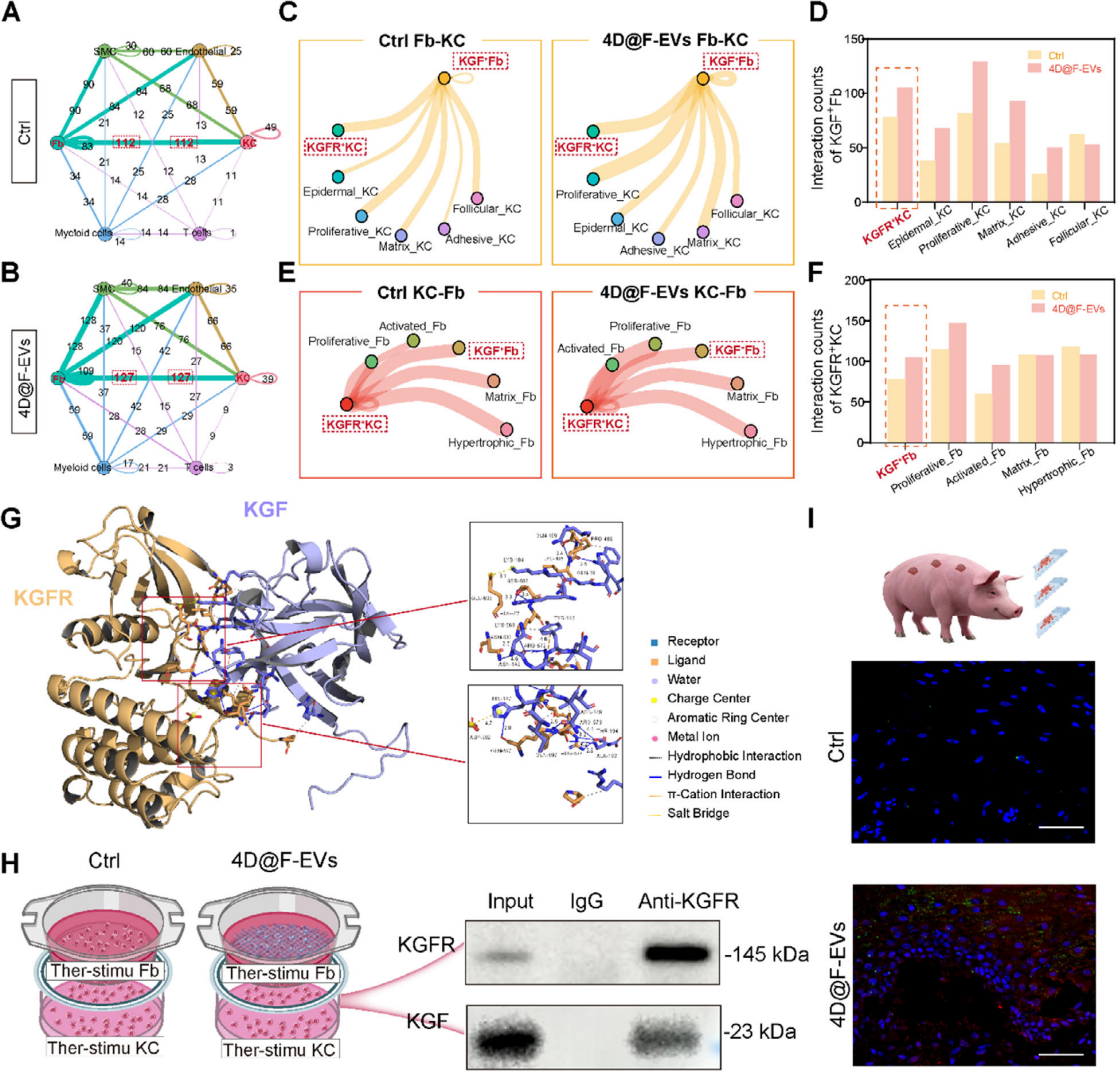
4D@F‐EVs modulate intercellular communication and function between KC and Fb. (A, B) Cell‐cells crosstalk networks for (A) 4D@F‐EVs and (B) Ctrl groups; line thickness indicates interaction strength. (C, D) Crosstalk network (C) and quantified communication events (D) linking KGF^+^Fb with all KC subtypes. (E, F) Crosstalk network (E) and quantified communication events (F) linking KGFR^+^KC with all Fb subtypes. (G) Molecular docking model of KGF–KGFR binding. (H) Co‐IP assay demonstrating the KGF–KGFR complex in heat‐stimulated KC co‐cultures. (I) Immunofluorescence images showing KGF (red) and KGFR (green) expression and localization in tissue sections from 4D@F‐EVs and Ctrl groups; scale bar: 200 µm.

Cell–cell communication networks were analyzed using the CellPhoneDB database based on scRNA‐seq data [31]. Compared with the control group, 4D@F‐EVs treatment markedly enhanced the crosstalk between Fb and KC (Figure 8A,B). To identify the key cellular subsets mediating Fb–KC interactions, the communication patterns between Fb subtypes and KC subtypes were further characterized. Among all subsets, KGF^+^ Fb and KGFR^+^ KC exhibited the most pronounced changes in cell–cell communication following 4D@F‐EVs administration, in addition to proliferative KC and general Fb (Figure 8C–F). The crosstalk between KGF^+^Fb and KGFR^+^KC may be mediated through the classical ligand‐receptor binding of KGF and KGFR [32]. Molecular docking calculations revealed that the binding free energy between KGF and KGFR was −59.2 kcal/mol and the docking score was ‐235.83, indicating the high affinity between KGF and KGFR. Specifically, KGF and KGFR could form hydrogen bonds (within 4.1A), salt bridges and Pi‐positive ion interaction, providing evidence for the KGF‐KGFR cell communication study (Figure 8G). Subsequent WB assays confirmed that the 4D@F‐EVs extract markedly upregulated KGF expression in Fb, whereas the rhFGF2 extract elicited a much weaker effect (Figure S23A; Figure S23B). This result indicated that the 4D‐CTH hydrogel, EVs and FGF2 exerted a remarkable synergistic effect in the 4D@F‐EVs system constructed in this study. In contrast, rhFGF2, without the microenvironmental support of this composite system, failed to effectively induce the significant high expression of KGF in Fb, thereby impeding its mediation of paracrine crosstalk between Fb and KC and the realization of their coordinated repair regulation.

Further, a co‐culture system consisting of heat‐stimulated Fb and heat‐stimulated KC was constructed. The expression of KGF and KGFR in the lysate of heat‐stimulated KC was detected. Moreover, Co‐IP analysis verified the physical interaction of KGF and KGFR (Figure 8H). Immunofluorescence staining additionally proved the significantly increased expression and clear co‐localization of KGF and KGFR in the wound tissue following 4D@F‐EVs treatment (Figure 8I). Together, these results further demonstrated that 4D@F‐EVs could promote the crosstalk between KGF^+^Fb and KGFR^+^KC via KGF‐KGFR receptor binding, thereby accelerating burn wound healing. Moreover, the interactions between *FGF2* and *FGFR1*, *FGFR2*, *FGFR3*, *FGFR4*, as well as with *FGF10*, *FGF22*, *FGF17*, etc., were also found to be strengthened (Figure S24A). Accordingly, the STRING database was employed to map the protein interaction network. Mechanistically, 4D@F‐EVs facilitate epidermal healing by stimulating KGFR^+^KC proliferation, driving epidermal regeneration, and exerting anti‐inflammatory effects, which are also regulated via the PI3K‐Akt pathway. Notably, crosstalk analysis between KGF^+^ Fb and KGFR^+^ KC revealed an intricate interaction network involving multiple *FGF* family ligands and *FGFR* family receptors (Figures S24B,C).

## 4 Discussion

**FIGURE 9:**
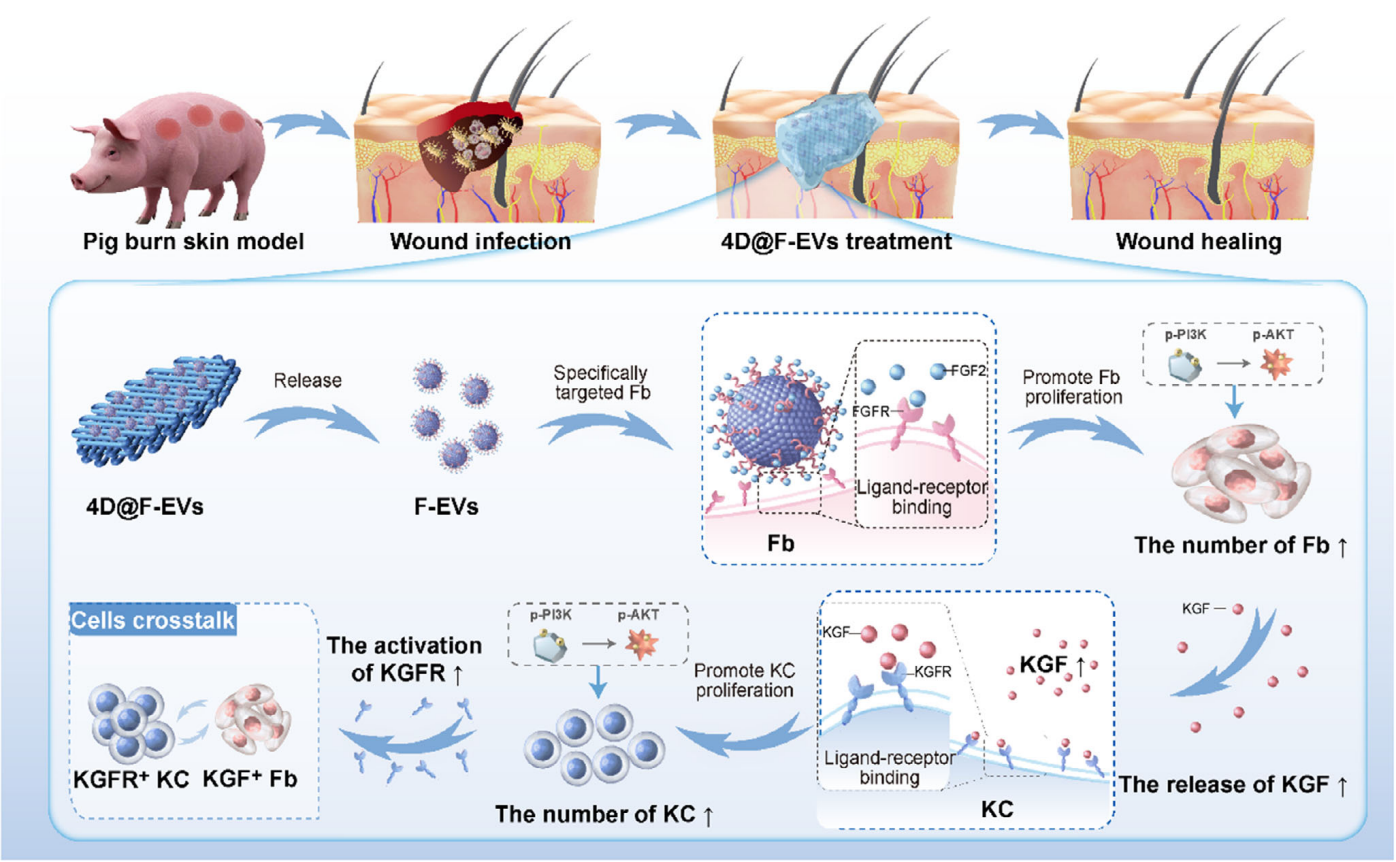
Molecular mechanisms underlying 4D@F‐EVs–mediated burn‐wound repair. F‐EVs released from 4D@F‐EVs deliver FGF2 that selectively engages FGFR on Fb, thereby activating the PI3K‐Akt signaling axis and markedly enhancing Fb proliferation. These expanded Fb secrete elevated levels of KGF, which in turn targets KGFR on KC, via the same PI3K‐Akt pathway, drives KC proliferation. KGF^+^Fb and KGFR^+^KC establish a positive‐feedback loop through cell‐cells crosstalk, generating a cascading amplification effect that ultimately accelerates wound healing.

To accelerate the synergistic crosstalk between Fb and KC in infected burn wounds, 4D@F‐EVs were engineered in this study to improve the targeting and therapeutic efficacy of FGF2, a key growth factor for burn repair. Our findings validate the feasibility and translational potential of 4D@F‐EVs for burn wound healing in large animal models. Furthermore, the regulatory mechanism underlying 4D@F‐EVs‐mediated burn tissue repair was systematically investigated and summarized (Figure 9).

Currently, research on ADMSC‐derived EVs (ADMSC‐EVs) primarily focuses on in vitro experiments and animal models. Their core mechanisms of action are centered on three main aspects: promoting of targeting cell proliferation, regulating local inflammatory response, and inducing angiogenesis [33, 34, 35, 36]. As research advances, multiple ADMSC‐EVs products have entered clinical development: A Phase II clinical trial demonstrated that ADMSC‐EVs combined with microneedling effectively improved facial photoaging symptoms, showing superior efficacy compared to platelet‐rich plasma therapy [37]. Additionally, an open‐label Phase I study confirmed the safety and tolerability of topically applied ADMSC‐EVs ointment for psoriasis treatment in healthy volunteers [38]. However, it must be objectively noted that large‐scale, multicenter randomized controlled clinical trials in humans remain scarce.

Regarding functional optimization strategies for ADMSC‐EVs, numerous studies have developed engineered EVs through techniques such as genetic modification, cellular preconditioning, or co‐delivery with carriers [39, 40]. Surface modifications or content regulation have significantly enhanced their autologous targeting and therapeutic efficacy [41]. Notably, studies loading FGF2 onto ADMSC‐EVs to achieve precise targeted delivery to Fb remain scarce to date. In addition to FGF2, other growth factors may also be considered for EV engineering in future studies, such as EGF, VEGF or PDGF, depending on the specific therapeutic goal. In principle, combining EVs with selected growth factors could further enhance epithelial regeneration, angiogenesis, or matrix remodeling. Nevertheless, such strategies require careful optimization, because wound repair is stage‐dependent and increasing formulation complexity may create additional challenges in manufacturing, stability control, and clinical translation.

Engineered F‐EVs fabricated via electrostatic adsorption offer a novel platform for the targeted delivery, signal recognition, and efficient transport of FGF2 using EVs as carriers. Positively charged modification of EVs by PDL allows stable binding of negatively charged FGF2 to the vehicle while preserving its biological activity. This charge‐based adsorption–desorption modification strategy can be extended to the loading and targeted delivery of other functional proteins, thereby expanding the potential for functional diversification of engineered EVs.

4D‐bioprinted technology played a key role in the delivery of engineered EVs and the protection of growth factor activity in this study. The 4D‐CTH scaffold fabricated via 4D bioprinting exhibited uniform porosity, customizable morphology, and temperature sensitivity, establishing a sterile, porous, and moist biomimetic microenvironment for engineered EVs. This system sustained the local release of EVs and their loaded growth factors at the injury site over an extended period [42]. Accordingly, 4D‐CTH not only furnished a favorable biomimetic niche for EVs but also mitigated the limitations of conventional FGF2 therapy—including high inactivation, short tissue retention, and limited duration of action in burn wounds —thereby substantially enhancing the therapeutic efficacy of FGF2 for burn treatment [27].

In vivo analysis and histological analyses have verified the satisfactory wound repair ability of 4D@F‐EVs in burn tissues of large animals. In the in vivo evaluation of the porcine skin burn model, the 4D@F‐EVs provided a wound healing rate of up to 98% for deep second‐degree burns within a 35 day treatment period, significantly outperforming other control groups and treatment groups. Histological assessment revealed that 4D@F‐EVs treatment exhibited more satisfactory physiological tissue healing characteristics at different time points after treatment, including accelerated epidermal regeneration and wound contraction, increased collagen fiber deposition in the dermis, promotion of new blood vessel formation, and hair follicle regeneration. These results confirmed that 4D@F‐EVs promote wound repair through cell proliferation promotion and improvement of the microenvironment, effectively enhancing the overall effect of burn treatment. Additionally, the organ pathological analysis after treatment confirmed the biological safety of 4D@F‐EVs, providing an important guarantee for the clinical translation of 4D@F‐EVs and reducing potential risks.

Most previous studies on burn treatment have focused on modulating specific cellular behaviors in target cells, yet have insufficiently investigated cell–cell crosstalk mechanisms underlying wound healing [43, 44, 45]. In this study, scRNA‐seq analysis of pig skin after burn injury was first applied to characterize the effects of 4D@F‐EVs on the cellular composition and transcriptomic profiles of wound‐related cells. Our results demonstrated that 4D@F‐EVs markedly enhanced the proliferation, migration and differentiation of Fb and KC by activating the PI3K‐Akt pathway, providing single‐cell‐level mechanistic evidence for the wound‐promoting effects of 4D@F‐EVs. Additionally, the scRNA‐seq analysis clarified the mechanism of cell‐to‐cell crosstalk between KGF^+^Fb and KGFR^+^KC in the wound healing process, offering robust insights for future investigations into the regulatory networks governing fibroblast and keratinocyte function within the wound microenvironment.

Although this study has achieved important progress in the application of engineered EVs for burn wound treatment, its clinical translation still faces multiple challenges. We fully acknowledge that a direct comparison with an FGF2+chitosan control group would further validate the additional advantages of EV‐mediated delivery strategies. The absence of this experimental design represents an important limitation of the present study. Notably, the core objective of this research was to establish a novel engineered EV‐based delivery platform, rather than to systematically compare different FGF2 delivery formats; therefore, this control was not included in the current work. Meanwhile, this comparative evaluation has been clearly defined as a key direction for future investigations, which will provide direct evidence for further verifying the unique superiority of the EV delivery system. In addition, further in‐depth stability assessments are still required: this study only confirmed that 4D@F‐EVs remain stable for 3 months at −80°C. Long‐term storage tests exceeding 3 months, as well as stability evaluations under variable humidity and transportation vibration conditions, are necessary to optimize storage and shipping protocols. Furthermore, scaling up manufacturing processes and formulating standardized clinical application guidelines are critical steps to promote the clinical translation of this technology.

## 5 Conclusion

In this study, engineered F‐EVs were successfully fabricated and integrated with 4D bioprinted thermoresponsive chitosan‐based hydrogel – 4D‐CTH to construct 4D@F‐EVs for the treatment of infected burn wounds. The combination of engineered extracellular vesicles and tissue‐engineered hydrogel carriers markedly improved the targeted delivery of growth factors and strengthened therapeutic efficacy. In the large animal model of infectious burns, 4D@F‐EVs treatment significantly accelerated wound healing by controlling infection and accelerating the signal transmission between KC and Fb. scRNA‐seq revealed that 4D@F‐EVs promoted KC and Fb proliferation via PI3K‐Akt activation and enhanced KC‐Fb crosstalk through the KGF‐KGFR axis, forming a positive feedback loop that facilitated epithelial regeneration and extracellular matrix remodeling. Collectively, 4D@F‐EVs represent an intelligent, precisely controllable, and highly effective therapeutic platform for infected burn wounds. This strategy demonstrates considerable potential for broad application and clinical translation across diverse therapeutic settings and routine skin care regimens.

## Author Contributions

Conceptualization: WH. X and XY. K. Methodology: XM. W and XY. L. Investigation: XM. W, XY. L, X. X, KL. Z, Z. S, NL. P, JL. L, YW. X, Y. T, L. Z, D. H, XT. L, TY. W, J. T, JX. S, MY. Z, X. M, Project administration: WH. X and XY. K. Writing – original draft: XM. W and XY. L. Writing – review and editing: WH. X, D. H and XY. K.

## Funding

This work was supported by Shandong Provincial Major Basic Research Project (ZR2026ZD35), Project of Marine Pilot Laboratory of Ministry of Science and Technology (10‐02); Shandong Taishan scholars special expert project (tstp20240824); National Key Research and Development Program (2022YEF0132500); Qingdao Natural Science Foundation (24‐4‐4‐zrjj‐150‐jch); National Natural Science Foundation of China(82302653); National Natural Science Foundation of China (Grant No. 32101137); National Natural Science Foundation of China (81770900); Horizontal Major Projects in Shandong Province (RH2200000157); Key Projects of Qingdao Science and Technology Department (20‐3‐4‐43‐nsh).

## Conflicts of Interest

The authors declare no conflicts of interest.

## Supporting information




**Supporting File**: advs77141‐sup‐0001‐SuppMat.docx.

## Data Availability

The datasets used and/or analysed during the current study are available from the corresponding author on reasonable request.
