# Supplementary material for: Application and Single‐Cell Regulation Mechanism of Engineered EVs Combined With 4D‐Printed Hydrogel for Infected Burn Repair
Source: Adv Sci (Weinh). 2026 Aug 21;13(62):e77141. doi: 10.1002/advs.77141 (PMC13496149; doi:10.1002/advs.77141)
Supplement: Supplementary file 1 [file ADVS-13-e77141-s001.docx]

**Supplementary Materials**

**1 Cell viability assay**

Calcein-AM/PI staining solution (Meilunbio, China) was adopted to detect cell viability. Cells were incubated with 2 μmol·L^-1^ AM solution and 8 μmol·L^-1^ PI solution at room temperature in the dark for 15 min. After the working solution was discarded, fluorescent images of stained cells were captured via an inverted fluorescence microscope.

**2 Cell Counting Kit-8 (CCK-8) assay**

CCK-8 (Meilunbio, China) was utilized to quantify cellular proliferative viability. Cells (5 × 10³ cells per well) were seeded into 96-well plates. After 36 h, the culture supernatant was aspirated, and cell monolayers were rinsed twice with PBS. Subsequently, 90 μL serum-free medium and 10 μL CCK-8 reagent were added. Absorbance values at 450 nm were measured at 0, 24, 48 and 72 h using a microplate reader (Bio-Rad, UK).

**3 Apoptosis assay detection**

Annexin V-FITC/PI Apoptosis Detection Kit (Meilunbio, China) was utilized for apoptosis assessment. Cells were digested with EDTA-free trypsin (Solarbio, China), and digestion was terminated with medium containing 10% fetal bovine serum (FBS). Cells were centrifuged (500 × g, 5 min) twice and resuspended in binding buffer to a concentration of 1×10⁶ cells·mL^-1^. A 100μL cell suspension was mixed with 5 μL Annexin V-FITC and 5μL PI, then incubated at room temperature in the dark for 15 min. Subsequently, 400 μL binding buffer was added for sample dilution, and fluorescent signals were measured on a flow cytometer (Beckman Coulter, China) to quantify apoptotic proportions.

**4 TEM transmission electron microscopy**

10 μL EVs solution was pipetted onto the copper mesh for 5 s and excess liquid was removed using filter paper. 10 μL 2% glutaraldehyde solution was pipetted onto the copper mesh for 5 min. The copper mesh was rinsed three times with PBS, each rinse lasting 1 min. 10 μL 2% phosphotungstic acid solution was pipetted onto the copper mesh for 1 min. After air-drying or nitrogen blow-drying, the specimen grid was observed under a transmission electron microscope (Veleta, Japan), and micrographs were acquired at an accelerating voltage of 80–120 kV.

**5 Western Blot**

Samples were lysed on ice for 30 min with high-strength RIPA lysis buffer supplemented with phosphatase and protease inhibitor cocktail (PMSF) (Solarbio, China). The samples were sonicated and centrifuged at 12, 000 rpm for 5 min. The supernatant was loaded onto sodium dodecyl sulfate polyacrylamide (SDS-PAGE) gels electrophoresis (Yeasen, China) with stem cell and extracellular vesicles protein samples and marker bands (Epizyme, China), and electrophoresed at 150 V for 40 min, and polyvinylidene difluoride (PVDF) membranes that had been activated for 5 min in anhydrous ethanol beforehand (Millipore, USA) was covered on an SDS-PAGE gel to assemble the sandwich structure, and the membrane was rotated at 400 mA current for 1 h.

After membrane transfer, the membrane was closed using rapid closure solution (Epizyme, China) for 15 min, and then incubated overnight at 4°C with specific primary antibodies: Alix (Proteintech, China, 1:5000), CD9 (Proteintech, China, 1:5000), TSG101 (Proteintech, China, 1:5000), CD63 (Proteintech, China, 1:5000), Calnexin (Proteintech, China, 1:5000), FGF2 (Abway, China, 1:1000), β-Actin (Abcam, UK, 1:4000), p-AKT (Cell Signaling Technology, USA, 1:1000), Akt (Cell Signaling Technology, USA, 1:1000), p-PI3K (Cell Signaling Technology, USA, 1:1000), PI3K (Cell Signaling Technology, USA, 1:1000).

Subsequently, membranes were washed multiple times with TBST buffer (Solarbio, China). mouse-derived secondary antibody (Affinity, China, 1:5000) and rabbit-derived secondary antibody (Yeasen, China, 1:10,000). The PVDF membranes were incubated at room temperature to allow specific recognition of the secondary antibody with the primary antibody-protein complexes, and finally, protein bands were detected using the Omni-ECL™ Ultrasensitive Chemiluminescence Detection Kit (Vazyme, China) and visualized using the Amersham ImageQuant™ 800 Protein Blotting Imaging System (Cytiva, Sweden). The bands were quantified using ImageJ software and GraphPad Prism 9.0.

**6 H&E staining**

At 7 days and 35 days post-burn, full-thickness skin tissues were harvested from a 2 cm region surrounding each porcine wound. The isolated tissues were fixed in 10% paraformaldehyde solution. After dehydration and paraffin embedding, 3 μm-thick paraffin sections were prepared and baked on a slide warmer at 60 °C for 1 h. The paraffin sections were then immersed in fresh xylene for three 10-min decerings. Sections were sequentially immersed in anhydrous ethanol, 95% ethanol, 80% ethanol, 75% ethanol, 60% ethanol, and distilled water for 5 min each. Subsequently, the sections were stained with hematoxylin solution for 2 min, washed with distilled water, and differentiated with acidic ethanol for 10 s, followed by washing with water for 30 s. Subsequently, the sections were rapidly dehydrated in 95% ethanol for 3 s, and subjected to three rounds of dehydration in absolute ethanol (10 min per cycle). After two 5-min immersions in xylene, the slices were mounted with neutral balsam mounting medium and air-dried. Finally, bright-field images were captured and analyzed using a panoramic digital slide scanner (3DHISTECH Pannoramic, Hungary).

**7 Masson staining**

Paraffin sections were stained with Weigert iron hematoxylin working solution (1:1 dilution) for 10 min, differentiated in acidic ethanol differentiating solution for 10 s, and rinsed with running water for 30 s. Subsequently, sections were blued with Masson’s bluing reagent for 5 min and washed with water for another 30 s. Then the slices were stained with xylidine ponceau solution for 30 s, followed by rinsing with weak acid working solution for 30 s. Next, sections were immersed in phosphomolybdic acid solution for 15 min and washed with weak acid working solution for 30 s. Afterwards, slices were transferred directly into aniline blue staining solution for 30 min, then rinsed again with weak acid working solution for 30 s. The sections were rapidly dehydrated in 95% ethanol for 3 s, followed by three rounds of dehydration in anhydrous ethanol (10 s per time). After two 5-min immersions in xylene, the slices were mounted with neutral balsam and air-dried. Finally, images were captured and observed via a panoramic digital slide scanner (3DHISTECH Pannoramic, Hungary).

**8 Sirius red staining experiment**

Paraffin sections were stained with Weigert iron hematoxylin working solution (1:1 dilution) for 10 min, differentiated in acidic ethanol differentiating solution for 10 s, and rinsed with running water for 30 s. Then the slices were incubated with Sirius Red mixed staining solution for 1 h, followed by rapid dehydration in 95% ethanol for 3 s. The sections were further dehydrated three times in anhydrous ethanol (10 s per cycle), then cleared twice in xylene solution (5 min each). After mounting with neutral balsam and air-drying, images were captured and observed using a panoramic digital slide scanner (3DHISTECH Pannoramic, Hungary).

**9 Immunohistochemical staining**

Slides were dewaxed via routine H&E dewaxing steps, then submerged in preboiled 1× sodium citrate antigen retrieval buffer. Samples were heated at medium power for 5 min; after replenishing the buffer, heating resumed for another 5 min. This cycle was repeated three times, and slides were cooled naturally to ambient temperature. Slides were blocked with BSA at room temperature for 30 min and rinsed thrice with PBS (30 s/wash). After endogenous peroxidase blockade (room temperature, 10 min) and three subsequent PBS washes, slides were incubated overnight at 4 °C with primary antibodies: CD31 (Proteintech, China, 1:2000) and α-SMA (Proteintech, China, 1:2000).

After three PBS rinses (30 s per wash), ready-to-use HRP-conjugated secondary antibody was applied and incubated at 37 °C for 20 min, followed by another three PBS washes. Sections were then incubated with DAB chromogen at room temperature; the chromogenic reaction was halted once brown-yellow precipitates emerged, prior to three further PBS rinses. Slides were counterstained with hematoxylin for 2 min and rinsed under running water, differentiated in acidic ethanol for 10 s, then washed with water for 30 s. Gradient dehydration was performed sequentially in 60%, 75%, 80% and 95% ethanol (5 min each), followed by two rounds of anhydrous ethanol treatment (5 min per cycle). After two xylene clearing steps (5 min each), slides were mounted with neutral balsam and air-dried, and images were acquired via a panoramic digital slide scanner (3DHISTECH Pannoramic, Hungary).

**10 Immunofluorescence staining**

Slides underwent identical dewaxing, antigen retrieval and blocking procedures as those for immunohistochemistry. Slides were incubated with primary antibodies at 4 °C overnight at the following dilutions: FGF2 (Abway, China, 1:100), FGF7 (KGF) (Abway, China, 1:200), FGFR2 (Affinity, China, 1:200). On the subsequent day, slides were rinsed three times with PBST (5 min per wash). Fluorophore-conjugated secondary antibodies were applied and incubated at 37 °C for 1 h, followed by three additional PBST washes (5 min each). After incubation in DAPI-containing anti-fade mounting medium for 5 min in the dark and air-drying, fluorescent images were captured using an Olympus IX73 inverted fluorescence microscope (Olympus, Japan).

**11 RNA extraction and Quantitative Real Time Polymerase Chain Reaction Analysis (qRT-PCR)**

Total serum RNA was extracted from normal and burned patients using TRIzol reagent (Takara, Japan). The RNA concentration in each sample was determined with a BioDrop spectrophotometer (Bio-Rad, USA). Following extraction, RNA was reverse-transcribed into cDNA using a stem-loop miRNA cDNA synthesis kit (Nanjing Novozan Biotechnology Co., Ltd., China). piRNAs cDNA was synthesized with a T100™ thermal cycler (Bio-Rad, USA). The expression levels of piRNAs were measured by SYBR Green I chimeric fluorescence, and qRT-PCR analysis was performed using the CFX Connect™ real-time system (Bio-Rad, USA). Gene expression was calculated using the 2^-ΔΔCT^ method, with β-actin serving as the internal control.

**12 Nanoparticle tracking analysis（NTA）**

The Zetasizer Nano S90 (Malvern Panalytical, Malvern, UK) was employed for EVs NTA analysis. After diluting the samples in PBS, five 30-second videos were captured by the camera. These videos were analyzed using NTA software 3.0 (Malvern Instruments Ltd), where the particle size distribution curve was integrated to determine the total particle number concentration.

**13 Zeta Potential**

A Zetasizer Nano ZS90 (Malvern Panalytical, Malvern, UK) was used to measure the zeta potential of EVs at a concentration of 0.01 mg/mL in PBS at 25°C.

**14 Antimicrobial experiment analysis**

**14.1 Bacterial inhibition rate test**

300 μL of 4D-CTH/4D@ F-EVs was placed in a 24-well plate, and 1 mL bacterial suspension (10^6^ CFU/mL) of *E. coli* (ATCC 25922, Gram-negative) and *S. aureus* (ATCC 6538, Gram-positive) was added. After incubation at 37°C for 24 h, the bacterial suspension was diluted 1,000-fold, and 1 μL suspension was evenly spread onto agar plates using the spread plate method. The plates were then inverted and placed in a bacterial incubator. After incubation at 37°C for 24 h, photographs were taken, and the number of colonies on each plate was counted to calculate the inhibition rate.

**14.2 Bacterial scanning electron microscope (SEM)**

Bacterial suspensions collected from the co-incubation system were centrifuged at 8000 × g for 5 min to harvest bacterial pellets, and the pellets were rinsed three times with PBS. The pellets were fixed in 2.5% glutaraldehyde at 4 °C for 4 h, followed by centrifugation and three additional PBS washes. Gradient dehydration was conducted sequentially in 30%, 50%, 70%, 80%, 95% and absolute ethanol (20 min for each concentration), and the absolute ethanol treatment was repeated three times. After mounting on silicon wafers and air-drying, bacterial morphological features were observed using a scanning electron microscope (Carl Zeiss, Germany).

**14.3 Live & Dead bacterial staining**

The Bacterial Live & Dead Staining Kit (DMAO/PI) (Beyotime, China) was used to assess bacterial viability. Take 100 µL of the co-incubated bacterial suspension, add 1 µL of DMAO solution and 1 µL of DMAO-PI solution, then incubate at 37°C in the dark for 30 min. Dispense 10 µL of the stained suspension onto a microscope slide. An inverted fluorescence microscope (Olympus, Japan) was used to capture images of the stained bacteria.

**14.4 Bacterial biofilm staining**

The ability of bacteria to form biofilms was assessed using the crystal violet staining method. *E. coli* and *S. aureus* were inoculated into TSB medium containing 2% glucose for cultivation. 0.1 mL aliquot of the bacterial suspension was transferred to a 24-well plate, supplemented with 0.9 mL of PBS or material extract, and incubated at 37°C for 48 h. The bacterial biofilm was harvested, washed three times with PBS, and fixed with methanol for 20 min. Methanol was discarded, and the sample was stained with 0.1% crystal violet solution for 15 min. After three PBS washes, the sample was photographed. To dissolve the crystal violet, 95% ethanol was added, and the mixture was gently shaken at room temperature for 15 min. An enzyme-linked immunosorbent assay reader (Bio-Rad, USA) was used to measure the absorbance at 595 nm.

**14.5 Endotoxin removal efficiency test**

Dynamic LAL colorimetry (Beyotime) was applied to assess endotoxin removal. A logT–logC standard curve (0.001–10 EU・mL^⁻¹^, R² ≥ 0.980) was calibrated. 4D@F-EVs and rhFGF2 gel were incubated with 0.1 EU・mL⁻¹ endotoxin solution at 37 °C for 1 h, followed by centrifugation to collect supernatants. After mixing with LAL reagent, absorbance at 405 nm was tracked every 30 s over 180 min at 37°C. Endotoxin clearance rate was calculated via the formula: [(Initial − Residual concentration) / Initial concentration] × 100%.

**Supplementary Table 1. Experimental Antibody Information**

| **Name** | **Brand** | **Source** | **Catalog Number** | **Dilution Ratio** | **Application** |
| --- | --- | --- | --- | --- | --- |
| CD63 | Proteintech | Wuhan, China | 67605-1-Ig | 1:5000 | WB |
| ALIX | Proteintech | Wuhan, China | 67715-1-Ig | 1:5000 | WB |
| TSG101 | Proteintech | Wuhan, China | 67381-1-Ig | 1:5000 | WB |
| CD9 | Proteintech | Wuhan, China | 60232-1-Ig | 1:5000 | WB |
| Calnexin | Proteintech | Wuhan, China | 66903-1-Ig | 1:5000 | WB |
| Human MSC Analysis Kit | BD biosciences | New Jersey, USA | 562245 | - | FCM |
| FGF7 (KGF) | Abway | Shanghai, China | CY8655 | 1:1000 | WB |
| FGF7 (KGF) | Abway | Shanghai, China | CY8655 | 1:200 | IF |
| CD31 | Proteintech | Wuhan, China | 11265-1-AP | 1:2000 | IHC |
| α-SMA | Proteintech | Wuhan, China | 14395-1-AP | 1:2000 | IHC |
| FGF2 (bFGF) | Abway | Shanghai, China | CY6691 | 1:1500 | WB |
| FGF2 (bFGF) | Abway | Shanghai, China | CY6691 | 1:100 | IF |
| FGFR2 | Affinity | Jiangsu, China | AF0159 | 1:1000 | WB |
| FGFR2 | Affinity | Jiangsu, China | AF0159 | 1:200 | IF |
| PI3K | Cell Signaling Technology | Danvers, USA | 4249 | 1:1000 | WB |
| p-PI3K | Cell Signaling Technology | Danvers, USA | 4228 | 1:1000 | WB |
| Akt | Cell Signaling Technology | Danvers, USA | 4091 | 1:1000 | WB |
| p-Akt | Cell Signaling Technology | Danvers, USA | 4060 | 1:1000 | WB |
| HRP Anti-beta Actin | Abcam | Cambridge, UK | AB20272 | 1:4000 | WB |
| Goat Anti-Mouse IgG (H+L) HRP | Affinity | Jiangsu, China | S0002 | 1:5000 | WB |
| Peroxidase AffiniPure Goat Anti-Rabbit IgG (H+L) | Yeasen | Shanghai, China | 33101ES60 | 1:10000 | WB |
| Goat Anti-Rabbit IgG (FITC) | Abway | Shanghai, China | AB0121 | 1:100 | IF |
| Goat Anti-Mouse IgG (FITC) | Abway | Shanghai, China | AB0122 | 1:100 | IF |
| Goat Anti-Rabbit IgG (AF594) | Abway | Shanghai, China | AB0151 | 1:100 | IF |
| Goat Anti-Mouse IgG (AF594) | Abway | Shanghai, China | AB0152 | 1:100 | IF |

**Fig. S1. Self-developed burn simulator.** (A) Core components of the self-developed burn simulator: 1: Main unit; 2: Wooden handle; 3: Metal rod; 4: Metal plate; 5: Temperature adjustment knob; 6: Power switch button; 7: Temperature display screen; 8: Pressure display screen; 9: Pressure adjustment push rod. (B) Physical diagram of the self-developed burn simulator.

**Fig. S2: Characterization of charge properties of EVs and PDL at different ratios.** (A) Nanoparticle tracking analysis (NTA) of EVs, with the total particle concentration calculated as the area under the particle size distribution curve (dilution ratio: 1:1000); (B) Zeta potential measurement results at different volume ratios of EVs to PDL (EV:PDL = 10:1, 5:1, 2:1, 1:1, 1:2).

**Fig. S3. Molecular docking of PDL with FGF2.** (A) Cartoon representation of the 3D interaction between the small molecule compound PDL (purple) and FGF2 (green). (B) 2D interaction between PDL and FGF2.

**Fig. S4. Relative Expression of FGF2 and EVs uptake of Fb.** (A) Relative protein expression levels of FGF2 (n=3). (B) Relative mean fluorescence intensity of PKH67 in Fb(n=3). Error bars represent M ± SD of triplicate experiments. **P* <0.05，***P* <0.01.

**Fig.S5. Co-IP verified the specific binding between FGF2 on F-EVs and FGFR1.**

 **Fig. S6. Effect of 4D-CTH and CH hydrogel extracts on L929 cell viability.** (A) Representative fluorescence images of live/dead cell staining showing L929 cell viability in the control group (Ctrl), 4D-CTH hydrogel extract group, and CH hydrogel extract group (green: live cells; red: dead cells). Scale bar: 200 μm; (B) Quantitative statistical analysis of cell viability in each group. Data are presented as mean ± standard error, *P < 0.05, ****P < 0.0001.

**Fig. S7. *In vitro* antibacterial activity and endotoxin characterization of 4D@F-EVs.** (A) Bacterial biofilm formation observed by crystal violet staining; (B) Quantitative analysis of OD values from crystal violet staining using a microplate reader; (C) Fluorescence staining images of live/dead bacteria with PI/DMAO. Green fluorescence (DMAO) labels all bacteria (live and dead), and red fluorescence (PI) labels dead bacteria with impaired membrane integrity. Scale bar: 100 μm; (D) Antibacterial rate analysis based on live/dead bacterial fluorescence staining; (E) SEM observation of bacterial morphology and membrane damage (pseudo-color). Green: *E. coli* with abnormal membrane structure; Red: *S. aureus* with abnormal membrane structure. Scale bar: 1 μm; (F) Statistical analysis of endotoxin clearance rate.

**Fig. S8. Stability of 4D@F-EVs under different storage conditions.** (A) SEM observation of hydrogel microstructure under various storage conditions; (B) Analysis of sample viscoelastic behavior via dynamic rheology testing (frequency sweep mode); (C) Detection of cumulative release characteristics of EVs; (D) Characterization of F-EVs' particle size distribution and concentration via NTA, with total particle number concentration defined as the area under the size distribution curve at a 1:500 dilution ratio; (E) WB was conducted to detect the expression levels of FGF2 protein in F-EVs.

**Fig. S9.** Fluorescence images of live/dead staining of Fb and Merged fluorescence intensity in 3D viewing; Green: live cell staining; Red: dead cell staining; Sacle bar: 100 μm.

**Fig. S10.** Fluorescence images of live/dead staining of KC and Merged fluorescence intensity in 3D viewing; Green: live cell staining; Red: dead cell staining; Sacle bar: 100 μm.

**Fig. S11. Biocompatibility characterization of 4D@F-EVs on normal and heat-injured cells.** (A) Viability of normal and heat-injured Fb and KC treated with 4D@F-EVs evaluated by live/dead cell staining (red: dead cells; green: live cells; scale bar: 200 μm). (B) Quantitative statistical analysis of cell viability. (C) Effect of 4D@F-EVs on the proliferation of normal and heat-injured Fb and KC dynamically monitored by CCK-8 assay at 0 h, 24 h, and 48 h.

**Fig. S12. Burn degree validation.** (A) Representative image immediately after burn. (B) H&E staining, scale bar 1000 μm (left), 200 μm (right). (C) Masson staining, scale bar 1000 μm (left), 200 μm (right). The dashed line indicates the boundary between the burned upper dermis and partial epidermis (above) and the unburned deeper dermis (below).

**Fig. S13. Biosafety of 4D@F-EVs.** (A) H&E staining of heart, liver, spleen, lung, and kidney in experimental and control groups. Scale bar: 200 μm.

**Fig. S14. UMAP clustering graph of the 4D@F-EVs and Ctrl.**

**Fig. S15. UMAP clustering graph of Fb of the 4D@F-EVs and Ctrl.**

**Fig.S16. KGF expression of KGF^+^Fb.** Differential expression of KGF among different Fb subpopulations in (A) UMAP and (B) violin graph; (C) KGF^+^Fb differentially expressed by KGF of 4D@F-EVs and Ctrl.

**Fig. S17. Enrichment analysis of Fb after 4D@F-EVs treatment.** (A) GO and (B) KEGG analysis.

**Fig.S18. 4D@F-EVs activated the PI3K-Akt pathway in KGF^+^Fb.** (A) GO and (B) KEGG analysis of significant different genes in KGF^+^Fb of 4D@F-EVs and control group; (C) GESA analysis of PI3K-Akt pathway.

**Fig. S19. UMAP clustering graph of KC of the 4D@F-EVs and Ctrl.**

**Fig.S20. KGFR expression of KGFR^+^KC.** Differential expression of KGFR among different KC subpopulations in (A) UMAP and (B) violin map; (C) KGFR^+^KC differentially expressed by KGFR of 4D@F-EVs and Ctrl.

**Fig. S21. Enrichment analysis of KC after 4D@F-EVs treatment.** (A) GO and (B) KEGG analysis.

**Fig.S22. 4D@F-EVs activated the PI3K-Akt pathway in KGFR^+^KC.** (A) GO and (B) KEGG analysis of significant different genes in KGFR^+^KC of 4D@F-EVs and Ctrl group; (C) Visualization of KEGG pathview with PI3K-Akt pathway.

**Fig. S23. 4D@F-EVs upregulates KGF protein expression in fibroblasts.** (A) Representative WB bands; (B) Gray value quantitative analysis of the corresponding bands. n=3 , *P < 0.05.

**Fig. S24. Protein regulatory networks of KGF⁺Fb and KGFR⁺KC.** (A) Ligand–receptor interactions between KGFR⁺KC and KGF⁺Fb; values indicate interaction strength. (B) PPI network revealing the intra-network regulation within KGF⁺Fb and KGFR⁺KC, as well as the inter-network crosstalk between KGF⁺Fb and KGFR⁺KC after 4D@F-EVs treatment. (C) Functional enrichment of key regulatory proteins involved in cell-cells interactions.
